# Polysaccharide-Based Nanocarriers for Natural Antimicrobials: A Review

**DOI:** 10.3390/polym17131750

**Published:** 2025-06-24

**Authors:** Elena Kotenkova, Aleksandr Kotov, Maxim Nikitin

**Affiliations:** 1 Moscow Center for Advanced Studies, Kulakova Str. 20, 123592 Moscow, Russia; alexkotov117@gmail.com (A.K.); max.nikitin@gmail.com (M.N.); 2 Department of Nanobiomedicine, Sirius University of Science and Technology, 1 Olympic Ave., Sirius Federal Territory, Krasnodar Region, 354340 Sochi, Russia

**Keywords:** chitosan, alginate, cellulose, gum, dextran, complexation, conjugation, essential oil, bacteriocins, AMP

## Abstract

Global concerns about environmental pollution, poor waste management, and the rise in antimicrobial resistance due to uncontrolled antibiotic use have driven researchers to seek alternative, multifaceted solutions. Plants, animals, microorganisms, and their processing wastes serve as valuable sources of natural biopolymers and bioactive compounds. Through nanotechnology, these can be assembled into formulations with enhanced antimicrobial properties, high safety, and low toxicity. This review explores polysaccharides, including chitosan, alginate, starch, pectin, cellulose, hemicellulose, gums, carrageenan, dextran, pullulan, and hyaluronic acid, used in nanotechnology, highlighting their advantages and limitations as nanocarriers. Addressing the global urgency for alternative antimicrobials, we examined natural compounds derived from plants, microorganisms, and animals, such as phytochemicals, bacteriocins, animal antimicrobial peptides, and proteins. Focusing on their protection and retained activity, this review discusses polysaccharide-based nanoformulations with natural antimicrobials, including nanoparticles, nanoemulsions, nanocapsules, nanoplexes, and nanogels. Special emphasis is placed on strategies and formulations for the encapsulation, entrapment, and conjugation of natural compounds using polysaccharides as protective carriers and delivery systems, including a brief discussion on their future applications, prospects, and challenges in scaling up.

## 1. Introduction

Antimicrobial resistance (AMR) has emerged as a critical challenge to modern healthcare systems worldwide. The uncontrolled use of antibiotics has been identified as a principal causative factor in the emergence of this critical public health challenge. Innovative antibacterial agents with unique modes of action are urgently needed to overcome current resistance challenges. Nanomaterials, natural antimicrobial compounds, and particularly their synergistic combinations offer promising solutions to address antimicrobial resistance [1,2,3,4].

Nanomaterials can be categorized into three primary classes based on composition: organic, carbon-based, and inorganic [5]. The key advantages of nanoparticles as carriers include their ultra-small and tunable size, protective encapsulation of payloads, controllable release kinetics, reduced systemic side effects, and capacity for combinatorial loading of therapeutic agents [6]. The utilization of nanocarriers as delivery systems enhances bioactive molecules’ therapeutic potential by improving their absorption, bioavailability, and stability while reducing toxicity and optimizing pharmacokinetic profiles [4].

Organic nanoparticles (NPs) fabricated from biopolymers exhibit favorable biocompatibility, biodegradability, and flexible loading capacity for various bioactive molecules [7,8]. Biopolymers are classified by origin as (1) synthetic polymers from bio-derived monomers (e.g., polylactic acid [PLA] and other polyesters), (2) natural animal- and plant-derived polymers (e.g., chitin, cellulose, collagen, and zein), and (3) microbial polymers (e.g., polyhydroxyalkanoates [PHA], bacterial cellulose, xanthan, and pullulan) [9]. Biopolymers directly extracted from biomass represent the most abundant category of natural polymers. This group encompasses polysaccharides (starch, cellulose derivatives, gums, alginates, pectins, and chitin/chitosan), animal proteins (casein, collagen, gelatin, and whey), and plant proteins (soy, pumpkin, wheat, and zein) [9,10,11,12,13], demonstrating significant potential for developing organic-based delivery systems [14]. Synthetic polymers, including those fabricated from bio-derived monomers, demonstrate several challenges in their application. The main limitations include potential toxicity, a complicated and expensive synthesis process, hydrophobicity, as well as poor biocompatibility and biodegradability of some synthetic polymers [15]. However, a group of synthetic polymers such as PLA, poly(lactide-co-glycolide) (PLGA), poly(caprolactone) (PCL), etc., are biocompatible and biodegradable [15,16]. PLA-based carriers have low drug loading capacity and encapsulation efficiency [17], while PLGA may develop biocompatibility problems [18].

**Figure 1:**
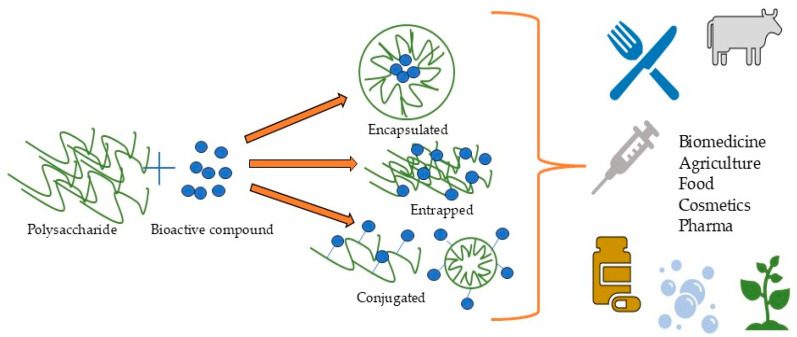
Formation of polysaccharide-based nanoformulations and their applications.

The combined factors of potential toxicity and immunogenicity aligned with the limited biocompatibility and accumulation risks of synthetic polymers combined with commercial interests have shifted focus toward biomass-derived biopolymers, which offer natural abundance and sustainable sourcing. Polysaccharides offer low immunogenicity and toxicity and excellent biocompatibility and biodegradability [19,20,21], and they are extensively utilized in biopolymeric nanoparticle fabrication [22,23], exhibiting not only superior mechanical and physicochemical properties but also intrinsic biological activity [24] that is often enhanced in nanoform. Bioactive compounds could be simply trapped, encapsulated, or conjugated with polysaccharides (Figure 1) [25]. Their nanoformulations as polysaccharide-based NPs can extend drug half-life, reduce cytotoxicity, and control release [26,27]. Polysaccharide-based nanoformulations serve as versatile delivery systems, material-reinforcing agents, and Pickering emulsion stabilizers for diverse applications including drug and gene delivery; antibacterial platforms; tissue engineering; cancer therapy; cosmetics; food fortification, preservation, and packaging; etc. [22,28,29,30,31].

Natural antimicrobials represent a diverse group of compounds derived from various sources, including plants, animals, and microorganisms [14]. These encompass phytochemicals (in either isolated forms or complex mixtures such as essential oils and extracts), peptides, proteins, polymers, etc. [32], and exhibit low toxicity, targeted antimicrobial activity (including against antibiotic-resistant strains), and high bioavailability [4,33,34]. Natural antimicrobial compounds face significant application challenges due to rapid degradation and volatility—issues that can be effectively addressed through encapsulation strategies [14].

This review systematically consolidates current knowledge on prevalent polysaccharides employed in nanocarrier synthesis, fabrication methodologies, and established nanomaterial technologies incorporating plant-, microbial-, and animal-derived antimicrobial compounds.

## 2. Natural Polysaccharides for Nanocarrier Fabrication

**Figure 2:**
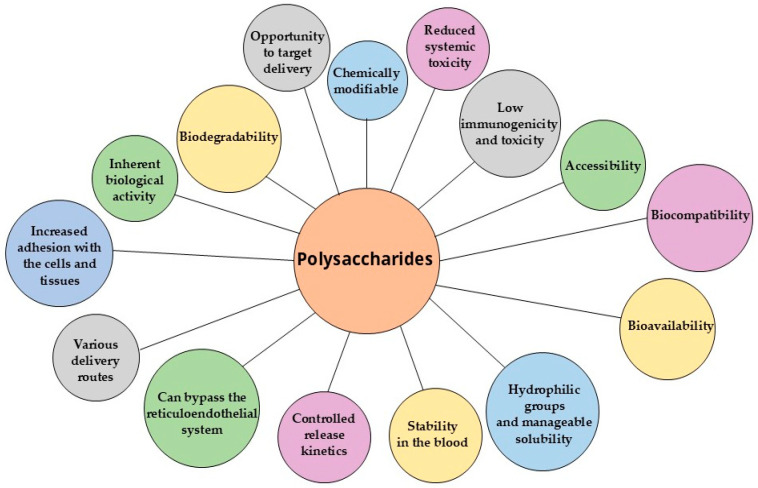
Benefits of polysaccharides as nanocarriers.

Polysaccharides are widely utilized in nanocarrier fabrication, either individually or in complex/conjugated forms [22,35,36,37,38,39,40]. Among natural polysaccharides, chitosan, alginate, and cellulose have been most extensively studied and implemented in nanoformulations [8,41,42]. Polysaccharide-based nanocarriers synergistically combine the advantageous properties of biopolymers, including low immunogenicity and toxicity, biodegradability, biocompatibility, and bioavailability, with the benefits of nanoformulations, such as enhanced drug bioavailability, prolonged drug half-life, reduced systemic toxicity, and controlled release kinetics [19,20,21,26,27,43]. Figure 2 illustrates the main common benefits of polysaccharides.

However, the toxicity and side effects of polysaccharide-based nanoformulations remain insufficiently characterized, necessitating further systematic studies to evaluate their potential adverse effects. Moreover, their properties could be influenced by a heterogeneous chemical structure, molecular weight, and modifications [44]. Table 1 presents the chemical structures of polysaccharides, and Table 2 systematically summarizes the non-common advantages and limitations of certain polysaccharides as nanocarriers.

Chitin (Table 1), a linear polysaccharide of poly(β-(1→4)-N-acetyl-D-glucosamine, is abundantly distributed in nature as crystalline microfibrils that constitute the structural framework of arthropod exoskeletons and fungal cell walls [45]. Industrially, chitin is sourced primarily from exoskeletons obtained as byproducts of global seafood processing [46,47]. Its extraction involves sequential treatments: (1) acid hydrolysis for demineralization (removing calcium carbonate), (2) alkaline solution for deproteinization, and (3) oxidative decolorization to yield a purified, colorless polymer [48,49].

Chitosan (Table 1), the principal chitin derivative, is produced via alkaline deacetylation [50]. Its physicochemical and biological properties are critically dependent on the molecular weight (50–2000 kDa), degree of deacetylation (54–100%), and functional group modifications (including nitration, sulfonation, graft copolymerization, and cross-linking) [51]. The deacetylation process constitutes a two-stage nucleophilic substitution reaction, typically achieved through thermal treatment in alkali at elevated temperatures [52,53]. Chitosan processing employs chemical, physical, and enzymatic techniques to achieve lower molecular weights, with physical methods being increasingly favored due to their ability to mitigate key limitations of alternative approaches—including random chain cleavage, high costs, and environmental concerns [54]. The most prevalent chitosan NP morphologies include nanospheres, nanocapsules, and nanofibers [55]. In recent decades, chitosan and its nanoforms have garnered significant research interest owing to their exceptional properties, particularly their biological activities: mucoadhesion, anti-inflammatory effects, antioxidant capacity, antimicrobial/antifungal action, antihyperglycemic activity, antitumor potential, and wound-healing promotion [55,56,57,58]. However, these materials may also exhibit cytotoxicity [51], which correlates with both acetylation degree and molecular mass [59].

Alginate, a highly abundant natural polysaccharide, exhibits unique cation-binding properties that enable the formation of stable and tunable hydrogels [60]. Alginate is primarily extracted from brown seaweed species and can also be produced through bacterial biosynthesis [61]. Alginate (Table 1) is composed of linear copolymer chains containing β-D-mannuronate (M) and α-L-guluronate (G) residues interconnected via 1→4 glycosidic linkages [62,63]. These monomers assemble into three distinct block configurations, poly-M homopolymers, poly-G homopolymers, and alternating MG heteropolymers, whose relative proportions dictate the polymer’s structural diversity, molecular weight distribution, and ultimately its functional physicochemical properties [64]. The molecular weight (32–400 kDa) and M/G composition of alginates are source-dependent, showing distinct variations across harvest locations and seasons [64,65]. Commercial alginate production employs both acidic and non-acidic extraction methods to isolate polysaccharides from brown seaweed biomass [65]. The classical acidic extraction method sequentially involves mineral acid treatment, alkaline conversion to sodium alginate, precipitation (CaCl_2_ or ethanol), and purification (acidification/Ca^2^⁺/ethanol) [64]. Novel extraction techniques are now being actively researched and implemented, including microwave-assisted extraction, ultrasound treatment, high-pressure processing, pressurized fluid extraction, and enzymatic hydrolysis methods [66]. Alginate-based nanocarriers (e.g., nanoparticles, nanofibers, nanoemulsions, nanocomplexes, and nanohydrogels) are extensively fabricated to encapsulate diverse bioactive payloads such as therapeutic drugs, proteins, and even whole cells [67,68]. Alginate nanomaterials exhibit advantageous physicochemical and biological properties, biocompatibility, pH sensitivity, mucoadhesiveness, and controlled biodegradability along with excellent safety profiles, though their performance is critically dependent on structural parameters (molecular weight, M/G ratio, and chemical modifications) [69,70].

Cellulose (Table 1) is a linear homopolysaccharide composed of D-glucoses connected by β(1→4) glycosidic bonds [71,72]. Cellulose is the most abundant renewable biopolymer on Earth, biosynthesized across multiple biological kingdoms including bacteria, plants, and even some animals [72]. The molecular weight of cellulose and its derivatives varies significantly with the degree of polymerization [73], which directly affects polymer solubility. Native cellulose is insoluble in conventional solvents due to its robust network of intermolecular and intramolecular hydrogen bonds, coupled with hydrophobic interactions [74]. Cellulose extraction can be performed across multiple scales (nano, micro, and macro) using chemical, mechanical, chemo-mechanical, or green techniques [75,76,77]. Both the mechanical and physicochemical properties of cellulose can be tailored through chemical modifications [78], though these processes typically require reagent application and subsequent purification steps to remove residual chemicals. Nanocellulose is broadly categorized into three principal types: cellulose nanocrystals (CNCs), cellulose nanofibrils (CNFs), and bacterial nanocellulose (BNC) [79]. The mechanical properties of cellulose-based materials critically determine their suitability for diverse applications [80]. Despite possessing unique advantages (high surface area, tunable surface chemistry, and exceptional mechanical strength), recent studies indicate that nanocellulose may exhibit complex toxicity profiles, necessitating further investigation into its biological safety [42].

Alongside cellulose, lignin and hemicellulose constitute fundamental components of plant cell walls, exhibiting remarkable properties. However, their inherent structural heterogeneity profoundly impacts all downstream processes, from extraction and purification to the final characteristics of derived nanomaterials [81,82]. Hemicellulose (Table 1) is a branched heteropolysaccharide composed of a sugar backbone with substituted side chains [83,84]. Hemicellulose comprises pentoses, hexoses, and uronic acids, including arabinose, xylose, glucose, mannose, galactose, glucuronic acid, and galacturonic acid, with minor constituents like fucose and rhamnose [85,86,87]. These form relatively short, branched chains of 500–3000 sugar units. In contrast, cellulose consists exclusively of linear β(1→4)-linked glucose polymers with higher degrees of polymerization (7000–15,000 units) [88,89]. The composition of this biopolymer varies significantly by species, with mannose-rich hemicellulose dominating softwoods and xylose-rich forms prevalent in hardwoods [90]. Hemicellulose is extracted from various plant sources and, while water-insoluble, can be isolated through alkaline treatment or mild acid hydrolysis [83,91]. Thus, conventional methods for hemicellulose extraction involve chemical treatments using alkali and organic solvents, while newer approaches employing H_2_O_2_, steam explosion, microwave, and ultrasonic techniques are also being investigated [92]. The mechanical, physical, chemical, and biological properties of hemicellulose vary significantly due to its structural heterogeneity and can be precisely manipulated through targeted modifications [93,94]. Current research indicates that nano-hemicellulose exhibits low toxicity and immunogenicity, though these properties remain insufficiently characterized in the literature [95].

Starch (Table 1), a plant-derived polysaccharide composed of amylose and amylopectin, is extracted from various agricultural crops [96]. Amylose is a linear α-glucan connected by α(1→4) glycosidic linkages, typically comprising 1000–20,000 glucose units with an average molecular weight of ~100 kDa [39,97,98]. Amylopectin is a highly branched macromolecule with a molecular weight of 1000–10,000 kDa, significantly larger than amylose. Its structure consists of α(1→4)-linked glucose chains with 5% α(1→6) branch points, creating a dendritic architecture [39,97,98]. Amylose typically comprises 5–35% of native starch, whereas genetically modified starches can achieve elevated amylose contents of 50–80% [99,100]. The amylose-to-amylopectin ratio significantly influences the physicochemical and functional properties of starch, with variations dependent on botanical origin [100,101,102]. Starch is stored in various plant organs, including seeds, fruits, roots, tubers, stems, and leaves [103,104]. Starch is primarily extracted through traditional methods involving washing, grinding, filtration, and sedimentation, typically via wet or dry processing pathways. Mechanical extraction methods additionally employ crushing, pressing, and centrifugation for higher yields [105]. Starch extraction is also accomplished through chemical methods (alkaline or surfactant-based treatments) and enzymatic approaches [106,107,108], with emerging green techniques gaining increasing application [109,110]. Native starch typically exhibits limited mechanical strength and poor water barrier properties [111,112]. Starch modification is crucial for enhancing functional properties and overcoming the inherent limitations of native starches [113]. Starch modification techniques include physical, enzymatic, and genetic methods [114]. Nanostarches are primarily categorized into two types: starch nanocrystals (SNCs) and starch nanoparticles (SNPs), which differ in their crystallinity and preparation methods [115,116]. Although starch nanoforms demonstrate enhanced mechanical strength, barrier performance, and thermal stability, these properties are strongly influenced by amylose/amylopectin ratios and often require modification to achieve optimal functionality [115,116,117,118,119].

**Table 2:** Benefits and drawbacks of polysaccharides as nanocarriers.

Biopolymer	Advantages	Disadvantages	Ref. and Pub. Year
Chitosan	✓ Source accessibility ✓ Chemically modifiable (customizable physical characteristics) ✓ Mucoadhesive ✓ Large surface area ✓ Free radical scavenging capability ✓ Hemostatic properties ✓ Antihyperglycemic effects ✓ Wound healing capabilities (activates macrophages and neutrophils, accelerates granulation tissue formation, promotes re-epithelialization, reduces scar formation and contraction, etc.) ✓ Antimicrobial and antifungal properties ✓ Anticancer activity ✓ Non-allergenic degradation intermediates ✓ Suitable for both hydrophilic and hydrophobic compounds	🗶 Properties influenced by MW and DA degree 🗶 Limited storage stability 🗶 High hydrophilicity 🗶 Poor solubility in aqueous solutions 🗶 Challenging extraction process (time-consuming, cost-intensive, requires substantial amounts of harsh chemicals) 🗶 Safety concerns related to polyanion type selection, technological factors (pH sensitivity, charge density, polymer concentration, requirements for organic solvents)	[51,55,58,59,120,121] 2023, 2023, 2023, 2024, 2022, 2025
Alginate	✓ Source accessibility ✓ Chemically modifiable (customizable physical characteristics) ✓ Hydrophilic (water-soluble) ✓ Mucoadhesive ✓ Capable of binding different cations, leading to stable and tailor-made hydrogels ✓ Cross-linking capability ✓ Antioxidant ✓ Anti-inflammatory ✓ Potential prebiotic activity ✓ Promotes wound healing ✓ Cell-affinitive ✓ Non-reactogenic ✓ Resistant to degradation in mammals ✓ Thermally irreversible ✓ Exhibits antibacterial and bacteriostatic properties through backbone negative charges or chelation capabilities	🗶 Properties influenced by heterogeneous chemical structure and MW 🗶 High hydrophilicity 🗶 Lacks processability via common techniques 🗶 Unclear cytotoxic effects due to impurities 🗶 Only low molecular weight alginate can be completely eliminated via renal system; longer macromolecules could be retained in circulatory system, inducing unexpected effects 🗶 Beneficial biological properties may be reduced during nanostructure fabrication 🗶 Stability under physiological-like conditions still poses significant doubts and pH-responsive swelling/contraction properties	[60,62,67,69,122,123] 2021, 2012, 2022, 2024, 2022, 2022
Cellulose	✓ Source accessibility ✓ Tunable surface chemistry ✓ Excellent mechanical strength (combining low density, high flexibility, strength, and chemical inertness) ✓ Thermally stable ✓ High aspect ratio ✓ High porosity ✓ Uniform material composition ✓ Crystalline structure with favorable rheological properties ✓ Unique three-dimensional (3D) network structure ✓ Excellent water retention and absorption ✓ Large surface area ✓ Capable of loading both charged and neutral drugs ✓ Enhances cell adhesion	🗶 Properties influenced by MW 🗶 Poor solubility 🗶 Crystallinity, surface morphology, surface chemistry, and dimensions are influenced by source and extraction technique 🗶 Often requires modification to achieve desirable properties 🗶 Safety concerns about required chemical reagents for modifications 🗶 Difficult to maintain crystal morphology after modification 🗶 Capable of cellular internalization, may cause inflammatory responses and induce oxidative stress 🗶 Low drug-loading capacity	[42,78,79,80,124,125] 2020, 2021, 2015, 2023, 2022, 2022
Hemicellulose and xylan	✓ Source accessibility ✓ Unique surface chemistry with tunable modifications ✓ High mechanical strength ✓ Gas and moisture resistant ✓ Excellent thermal stability ✓ Large surface area ✓ High aspect ratio ✓ Solid crystalline structure and amorphous branching architecture ✓ Supports cell growth and proliferation ✓ Antibacterial properties ✓ Protein and biomolecule interaction capability ✓ Stimuli-responsive behavior ✓ UV radiation absorption	🗶 Properties influenced by heterogeneous chemical structure and MW 🗶 Poor solubility 🗶 Physicochemical characteristics depend on source and extraction technique 🗶 Often requires modification to achieve desirable properties 🗶 Safety concerns about required chemical reagents for modifications 🗶 Difficult to modify due to heterogeneity	[86,92,93,95,126,127] 2024, 2020, 2021, 2023, 2021, 2024
Starch	✓ Source accessibility ✓ Chemically modifiable (customizable physical characteristics) ✓ Hydrophilic (water-soluble) ✓ Large surface area ✓ Superior absorption capacity ✓ Diverse morphological forms ✓ Highly reactive surface ✓ Excellent biological penetration rate ✓ Digestible	🗶 Properties influenced by amilose/amilopectin ratio 🗶 Limited storage stability 🗶 High hydrophilicity 🗶 Often requires modification to achieve desirable properties 🗶 Safety concerns about required chemical reagents for modifications 🗶 Retrogradation tendency 🗶 High viscosity at low concentrations 🗶 Poor freeze–thaw stability	[115,116,117,118,119] 2020, 2023, 2022, 2015, 2024
Pectin	✓ Source accessibility ✓ Chemically modifiable (customizable physical characteristics) ✓ Hydrophilic (water-soluble) ✓ High water absorption and moisture retention ✓ Cross-linking capability ✓ Large surface area ✓ Dual encapsulation (hydrophilic and lipophilic compounds) ✓ Drug-conjugating (forms covalent bonds) ✓ Mucoadhesive ✓ Antioxidant ✓ Anti-inflammatory ✓ Probiotic activity ✓ Anticancer ✓ Stimulates B-cell proliferation and macrophage IL-1β secretion ✓ Targets liver cancer (via galactose receptors) ✓ Gastrointestinal stability (useful for colon-targeted delivery)	🗶 Properties influenced by heterogeneous chemical structure, DE/DA degree, and ratio of pectin types 🗶 High hydrophilicity 🗶 Digestive interference	[128,129,130,131,132,133] 2023, 2015, 2008, 2021, 2023, 2022
Gums	✓ Chemically modifiable (customizable physical characteristics) ✓ Hydrophilic (water-soluble) ✓ High water absorption and moisture retention ✓ Reactive-site-rich (for molecular interactions) ✓ Thermostable ✓ pH-stable (wide range) ✓ Mucoadhesive ✓ Antioxidant ✓ Immunomodulatory ✓ Antibacterial properties ✓ Gastrointestinal stability and microbially degradable in intestinal conditions (useful for colon-targeted delivery)	🗶 Properties influenced by heterogeneous chemical structure and MW 🗶 High hydrophilicity 🗶 Unpredictable hydration kinetics	[134,135,136,137] 2020, 2023, 2019, 2025
Carrageenan	✓ Chemically modifiable (customizable physical characteristics) ✓ Tunable solubility (sulfo groups) ✓ Electrolyte- and temperature-dependent chain conformation ✓ Anticoagulant properties ✓ Anticancer activity ✓ Immunomodulatory effects ✓ Antioxidant capacity ✓ Antibacterial/antiviral potential ✓ Supports cell adhesion/proliferation ✓ Enzyme-degradable (via glycosidic bond hydrolysis) ✓ Polyelectrolyte behavior (sulfate group-mediated)	🗶 Properties influenced by variable chemical structure (e.g., sulfate group content) and MW 🗶 Organic solvent insolubility 🗶 Physicochemical characteristics depend on source and extraction technique 🗶 Functionality loss under extreme conditions (high temperature/low pH)	[138,139,140,141,142,143,144,145,146] 2021, 2022, 2021, 2017, 2017 2001, 2023, 2013, 2017
Dextran	✓ Hydrophilic (water-soluble) ✓ Chemically modifiable (customizable physical characteristics) ✓ Simple glucan structure (repetitive units) ✓ Cross-linkable ✓ pH-stable (mild acidic/basic conditions) ✓ pH-responsive tunability (via chemical modification) ✓ High drug-loading capacity ✓ Antioxidant and radical scavenging ✓ Organ degradation (by liver/spleen/kidney/colon dextranase) ✓ Safe metabolic byproducts (enzyme-digested)	🗶 Properties influenced by MW 🗶 Limited storage stability 🗶 Synthesis concerns (impurities during isolation and purification) 🗶 May require modification to achieve desirable properties 🗶 Safety concerns about required chemical reagents for modifications 🗶 Potential side effects: volume overload, pulmonary edema, platelet dysfunction, cerebral edema, anaphylaxis	[43,147,148,149,150,151] 2023, 2022, 2016, 2022, 2023, 2020
Pullulan	✓ Hydrophilic (water-soluble) ✓ Chemically modifiable (customizable physical characteristics) ✓ Capable of hydrophobic drug conjugation/complexation ✓ Good moisture retention ✓ Thermostable ✓ Responsive to stimuli (pH, temperature, and light) ✓ Adhesive ✓ Enhances permeation ✓ Antioxidant ✓ Anti-inflammatory ✓ Immunomodulatory ✓ Fungal growth inhibition ✓ Anticancer activity ✓ Liver-targeting affinity ✓ Effective for percutaneous and transmucosal protein delivery ✓ Non-mutagenic ✓ Undergoes glycosidic hydrolysis and glucose metabolism ✓ Liver-targeting affinity ✓ Slowly degraded by microbiota	🗶 Properties influenced by MW 🗶 Limited storage stability (lacks inherent antibacterial properties) 🗶 High hydrophilicity 🗶 May require modification to achieve desirable properties 🗶 Safety concerns about required chemical reagents for modifications 🗶 May cause mild gastrointestinal discomfort	[152,153,154,155,156,157,158,159,160] 2022, 2021, 2021, 2016, 2016, 2013, 2023, 2023, 2025
Hyaluronic acid	✓ Hydrophilic (water-soluble) ✓ Chemically modifiable (customizable physical characteristics) ✓ Good moisture retention ✓ Immune-stimulating and angiogenic (4–200 kDa) ✓ Involved in wound healing and embryogenesis (200–500 kDa) ✓ Antimicrobial/anti-inflammatory/antiangiogenic (>500 kDa) ✓ Ligand for cell surface receptors/CD44-targeting moiety ✓ Transcutaneously administrable ✓ Anti-edematous ✓ Analgesic ✓ Antioxidant ✓ Anti-adhesive ✓ Tissue-regenerative ✓ Drug retention time-prolonging ✓ Gastroprotective and intestinally-releasing ✓ Hyaluronidase-degradable	🗶 Properties influenced by MW 🗶 Limited storage stability 🗶 High hydrophilicity 🗶 Batch variability (source- and process-dependent) 🗶 Potential immunogenicity (especially modified forms) 🗶 Poor hydrophobic drug loading 🗶 Molecular weight limits tissue penetration	[161,162,163,164,165,166] 2020, 2022, 2024, 2024, 2021, 2022

Notes: MW—molecular weight; DA—deacetylation degree; DE/DA—degree of esterification and amidation (DA).

Pectins are hydrophilic polymers naturally present in plant cell walls [167]. Their functional properties are determined by the degree of esterification (DE) and degree of amidation (DA) [130]. Pectins consist of a covalently linked, galacturonic acid-rich polysaccharide backbone (up to 70%, α-1,4-linked) and are classified into the following main types: homogalacturonan (HG, Table 1) as the major component, and rhamnogalacturonan I (RGI), rhamnogalacturonan II (RGII), xylogalacturonan (XGA), and apiogalacturonan (AGA) as the minor components [168,169,170]. The backbone of RGI contains L-rhamnose, while the branched structures of pectins (RGI, RGII, and XG) include L-rhamnose, L-arabinose, D-apiose, D-glucuronic acid, D- and L-galactose, D-xylose, L-fucose, and L-aceric acid [171]. Pectin is commercially extracted from sugar beet pulp, citrus peel, and apple pomace through conditional extraction with mild acids, as well as enzyme-, microwave-, and ultrasound-assisted extraction, subcritical water extraction, pressurized CO_2_ and deionized water methods, deep eutectic solvents, and ohmic heating [171,172]. Pectin exhibits poor mechanical properties and moisture resistance [133]. Pectin-based nanostructures, including nanoparticles, nanoemulsions, nanocapsules, and nanogels, have attracted significant interest due to their outstanding properties, such as physical sensitivity (to light, temperature, and electricity), chemical sensitivity (to pH, redox reactions, and glucose), and biological sensitivity (to enzymes) [173,174].

Plant gums are polysaccharides composed of covalently bound sugar monomers [134]. The most extensively studied varieties include arabic gum, carrageenan, xanthan gum, and tragacanth [136]. Their structural backbones typically contain galactose, arabinose, rhamnose, uronic acids, galacturonic acid, proteins, calcium, and magnesium [175]. These biopolymers exhibit unique structural and biological properties—including water retention capacity, thermal stability, and hydrophilicity, along with antibacterial, antioxidant, and immunomodulatory activities—making them promising candidates for drug delivery applications [137]. While less studied than other biopolymers, gum-based nanostructures such as nanoemulsions, nanoparticles, nanocomplexes, and nanofibers have shown particular promise for colorectal therapy due to their selective digestibility properties [134,135,136]. Carrageenan (Table 1) is extracted from specific red seaweed species through alkaline processing [176]. This sulfated polysaccharide consists of alternating galactose and anhydrogalactose units linked by glycosidic bonds [177], with an average molecular mass exceeding 100 kDa. The major types of carrageenans are ι (iota), κ (kappa), and λ (lambda), which differ in their sulfate group content [178,179]. Carrageenan exhibits significant antioxidant, immunomodulatory, and disease-preventive properties, making it a promising candidate for pharmaceutical development [144]. Its chain conformation and gelation behavior are strongly influenced by temperature and the electrolyte concentration [139], but when carrageenan is used as an individual matrix material, zero-order kinetics and pH-independent release profiles cannot be achieved [146]. Toxicological studies indicate excellent biocompatibility with no observed teratogenic effects [145].

Dextran (Table 1) is a neutral bacterial exopolysaccharide produced by lactic acid bacteria or their enzymes in sucrose-rich environments, featuring α-(1→6)-linked glucose backbones with potential α-(1→2), α-(1→3), or α-(1→4) branches, and exhibiting molecular weights ranging from <40 kDa (low) to >40 kDa (high) [148,180,181]. The solubility and rheological properties of dextran depend on its molecular weight (up to 440 MDa) and branching structure [180]. Dextran’s alcohol insolubility enables ethanol/methanol precipitation (the conventional isolation method), with contemporary approaches employing membrane filtration or deep eutectic solvents [182,183]. The polymer’s molecular weight determines the functionality of dextran-coated NPs and bioconjugates [184,185]. While comprehensive toxicity profiles remain scarce [149], dextran’s optimal biocompatibility and tunable properties [150] have established it as a premier nanocarrier material [186], adaptable to nanoparticle, nanogel, microsphere, and micelle formulations [151].

Pullulan (Table 1) is a natural, neutral exopolysaccharide primarily composed of maltotriose units, produced through aerobic fermentation by black yeasts [152,187]. Maltotriose units consist of three glucose molecules linked by α-1,4 glycosidic bonds, which are further connected to each other via α-1,6 glycosidic bonds [188,189]. The molecular weight of pullulan ranges from 45 to 600 kDa depending on the culture conditions and the strain of *A. pullulans* [190], and it significantly influences its rheological and mechanical properties [191]. The production of pullulan involves microbe harvesting, the elimination of unwanted substances (mainly melanin), precipitation, membrane purification, and freeze drying [164]. Pullulan is virtually insoluble in organic solvents; thus, ethanol and isopropanol are the most commonly used solvents for its purification. However, chromatographic techniques or aqueous two-phase systems may also be employed [192]. Pullulan exhibits high chemical versatility, enabling facile modification through carboxymethylation, oxidation, amination, sulfation, acetylation, and esterification [153,158]. Hydrophobic modifications, in particular, facilitate the spontaneous self-assembly of nanocarriers, which exhibit high drug-loading capacity and a low critical micellar concentration, making them promising for targeted delivery systems [153,158]. Pullulan-based nanostructures can be engineered into diverse forms, including nanoparticles, nanogels, and nanoplexes, leveraging the enhanced permeability and retention effect [154]. Despite its advantages, pullulan’s poor mechanical strength, extreme hydrophilicity, and lack of inherent antibacterial properties restrict its applications [193].

Hyaluronic acid (HA, Table 1) is an anionic polysaccharide consisting of D-glucuronic acid and N-acetyl-D-glucosamine linked by β-1,3- or β-1,4-glycosidic bonds [37]. It is produced by membrane-bound HA synthases and can be obtained through extraction from animal tissues, microbial production, or cell-free enzymatic synthesis [166], with membrane technologies being the preferred purification technique [194]. The molecular weight of HA, which can exceed 500 kDa, significantly influences its structural, biological, physical, physicochemical, and degradation characteristics [163,195]. HA functions as an endogenous ligand for cell surface receptors, with high affinity for CD44. This receptor-specific binding enables HA-based systems to selectively target and deliver therapeutic agents to pathological tissues [165]. Despite its outstanding properties, HA has several substantial limitations. These include rapid biodegradation, poor stability and mechanical strength, extreme hydrophilicity, limited ability to encapsulate and deliver hydrophobic drugs, molecular weight-dependent tissue penetration, and potential immunogenicity, particularly for chemically modified forms. Many of these limitations can be addressed through strategic chemical modifications [161,164].

Polysaccharides are renewable, widely accessible, biodegradable, and biocompatible materials that confer low toxicity and allergenicity to polysaccharide-based nanomaterials. Due to their unique physical and chemical properties, polysaccharide-based nanocarriers exhibit all advantageous characteristics of organic nanocarriers, including an extended drug half-life, enhanced solubility, and controlled release profiles. Furthermore, these nanocarriers can be readily modified to achieve tailored properties while inherently possessing various beneficial biological activities. The main limitation in applying polysaccharides for nanoformulation is their variable chemical structure, which affects physicochemical properties and water absorption capacity, along with poorly characterized cytotoxic effects that may arise from structural variations, modifications, impurities, or NP characteristics (size, shape, and biological barrier penetration ability) [37].

## 3. Methods of Synthesis for Polysaccharide-Based Nanocarriers

Polysaccharide nanocarriers can be synthesized through various methods including self-assembly, ionic gelation, cross-linking, complex coacervation, emulsification, ultrasonication, desolvation, supercritical fluid technology, and nanoprecipitation or solvent displacement, with the choice of method depending on the desired nanoparticle characteristics and polysaccharide properties [36,196].

**Figure 3:**
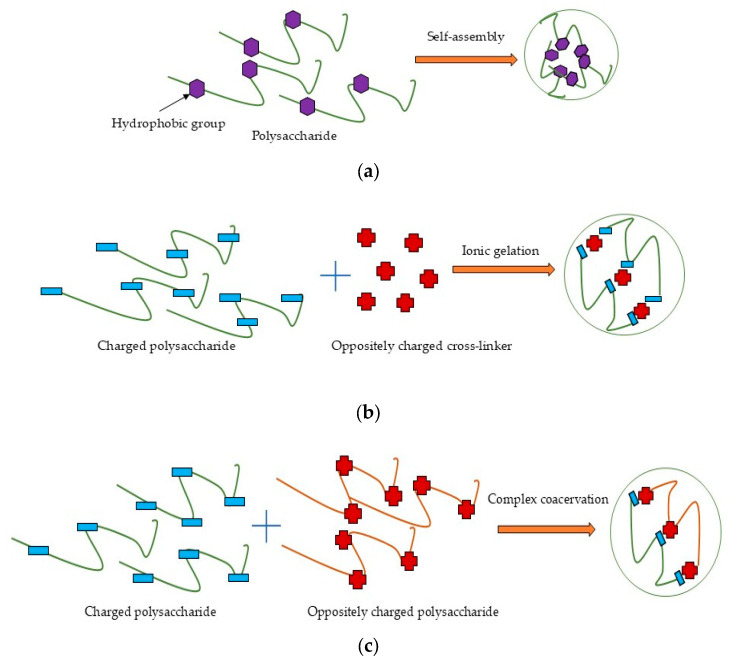

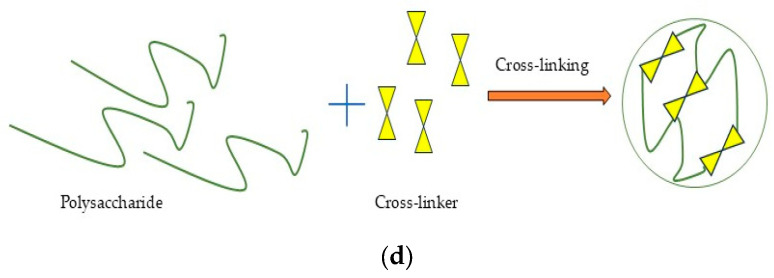
Schematic illustration of preparation process for polysaccharide nanoformulations: (**a**) self-assembly; (**b**) ionic gelation; (**c**) complex coacervation; (**d**) cross-linking.

Self-assembly (Figure 3a) is one of the most promising techniques for nanoparticle synthesis due to its environmental friendliness, biocompatibility, process simplicity, and low toxicity [40]. This method involves the spontaneous organization of amphiphilic polysaccharides into ordered nanostructures with a hydrophobic core (for drug encapsulation) and a hydrophilic shell (for aqueous stability). The driving forces include non-covalent interactions such as hydrophobic effects, electrostatic forces, van der Waals interactions, and π–π stacking (in aromatic-modified systems) [36,40,197]. Chemical modification (e.g., grafting hydrophobic chains) can convert naturally hydrophilic polysaccharides into amphiphilic polymers, enabling controlled self-assembly [36]. Conversely, chemical modification involves the application of harmful reagents and could be inefficient and wasteful [198], therefore requiring subsequent purification steps, and it limits the potential of self-assembly for the industrial scale.

Ionic gelation (Figure 3b) is a widely used method to form nanostructures from charged polysaccharides through electrostatic interactions with oppositely charged cross-linkers in dilute aqueous solutions [22]. Chitosan and other cationic polysaccharides can be cross-linked with polyanions like tripolyphosphate (TPP), whereas anionic polysaccharides such as alginate are typically cross-linked with cations like Ca^2^⁺ from CaCl_2_ [199,200,201]. Ionic gelation is a widely used nanoparticle synthesis method characterized by its simplicity and convenience. Furthermore, this controllable process avoids organic solvents and exhibits no toxicity [36] and can thus be used for industrial scale-up by optimizing the polymer and cross-linker concentration and mixing speed or by using droplet-producing devices [202,203].

Complex coacervation (Figure 3c) occurs through electrostatic interactions between oppositely charged species, typically involving two oppositely charged polysaccharides [36,204]. Electrostatic complexation induces phase separation, forming a biopolymer-rich coacervate phase and a solvent-rich phase [205]. Ionic strength, pH, temperature, charge density, and polyelectrolyte molar mass critically influence coacervate particle stability and formation, while these systems also exhibit excellent biocompatibility and low toxicity [22]. The simplicity and safety of this method made it attractive for scaling up, but it requires careful consideration of numerous factors like mixing systems, polymer ratios and concentrations, temperature, pH, and balance in ionic strength [206]. 

Covalent cross-linking (Figure 3d) in the nanoformulations created irreversible cross-linking points, yielding highly stable nanostructures [22]. Glutaraldehyde, compounds with PO_4_^3−^ groups, enzymes, carbodiimide, genipin, epoxy, acrylamide, citric acid, formaldehyde, etc., are the most common synthetic and natural cross-linkers applied for polysaccharides [207]. The application of toxic reagents in covalent cross-linking has limited its industrial scalability. To address this challenge, green alternatives including citric acid, tannic acid, vanillin, gallic acid, ferulic acid, proanthocyanidins, phytic acid, squaric acid, and epigallocatechin have been investigated for developing polysaccharide-based nanoformulations [208].

**Figure 4:**
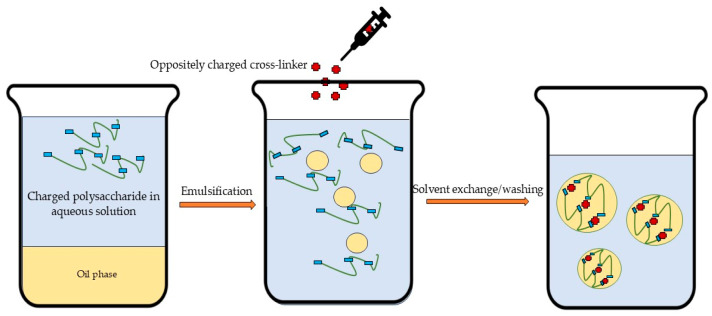
Schematic illustration of emulsification followed by ionic gelation.

Emulsification (Figure 4) can be performed in either oil-in-water or water-in-oil systems through mechanical stirring or ultrasonication [36]. More complex systems, such as double emulsions (oil-in-water-in-oil or water-in-oil-in-water), may also be employed for specialized applications [22]. Ultrasound treatment can depolymerize biopolymers, modify their physical properties (e.g., viscosity and molecular weight), and promote structural reorganization of polysaccharide molecules [209,210]. Nanoparticles can be obtained from emulsions through various methods including internal/external gelation, solvent evaporation/diffusion, and reverse salting out [22]. Despite enabling the production of controllable, spherical nanoparticles, emulsification remains a more complex technique that requires significantly greater energy input and larger quantities of organic solvents compared to alternative methods [25,36,56]. These requirements make scale-up more complicated and energy-intensive, and consequently more expensive. Additionally, the technique requires the optimization of numerous parameters, including ones for internal/external gelation, solvent evaporation/diffusion, and reverse salting out [25].

**Figure 5:**
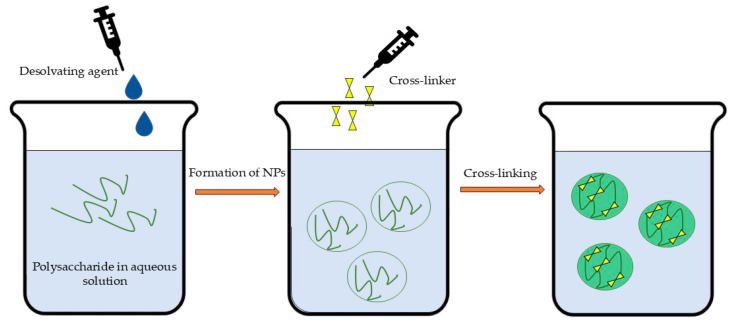
Schematic illustration of desolvation followed by cross-linking.

The desolvation technique (Figure 5) involves the coacervation or precipitation of dissolved polysaccharides through the gradual addition of desolvating agents (e.g., salts and alcohols). This process is typically followed by cross-linking to stabilize the formed nanoparticles [38,211]. The selection of desolvating agents depends on the encapsulated substance’s properties [22] but may require a subsequent purification step due to the potential toxicity of residual molecules [36], which could limit its scaling-up ability. The desolvation method is typically used for protein nanoparticle preparation, but there is a lack of systematic studies on this approach for polysaccharides, particularly regarding physical stability [212]. However, the desolvation technique followed by cross-linking could be successfully applied for the fabrication of polysaccharide NPs [213].

**Figure 6:**
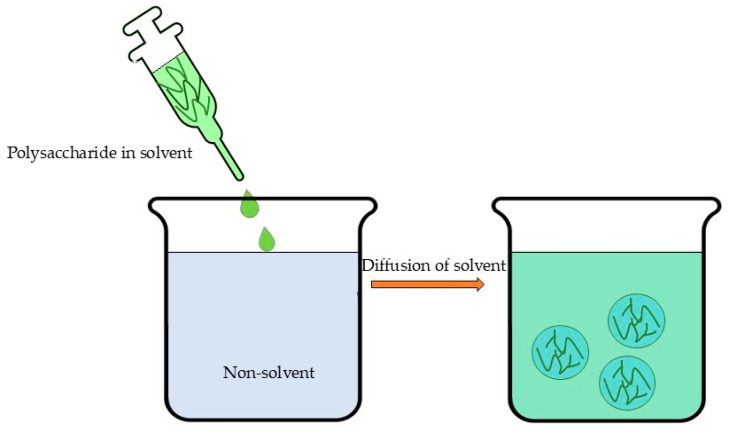
Schematic illustration of nanoprecipitation or solvent displacement.

Nanoprecipitation or solvent displacement (Figure 6) is an easy, less complex, less energy-consuming, widely applicable method that involve the following steps: (1) the dissolution of the polymer in a fully or partly water-miscible solvent, (2) the transfer of the solution to another non-solvent which may contain a surfactant, and (3) nanoprecipitation due to the rapid diffusion of solvent provided that aggregation is limited [214,215,216]. The excess solvent is typically eliminated through evaporation, dialysis, or lyophilization [22]. However, this method’s primary limitations involve both the requirement for organic solvents and their substantial dilution to prevent particle aggregation during precipitation [36]. Furthermore, studies indicate that nanoprecipitation demonstrates optimal efficacy for hydrophobic drugs [217]. These limitations, coupled with the demanding optimization requirements, present significant challenges in scaling up nanoprecipitation and solvent displacement methods for industrial applications.

Equipment-based methods such as electrospinning enable the fabrication of polysaccharide nanomaterials with enhanced specific surface area and porosity [218]. However, challenges in electrospinning polysaccharides include poor solubility (e.g., cellulose), high viscosity, and elevated surface tension [219]. These limitations can be addressed through optimized solvent selection, material modification, and tailored structural design [220].

Target substances can be incorporated into nanostructures either through covalent conjugation via surface functional groups or via physical encapsulation during fabrication, mediated by electrostatic interactions, hydrogen bonding, or other entrapment mechanisms [196,221,222].

Safety concerns associated with toxic or potentially toxic reagents and challenges in process parameter optimization represent major barriers to industrial-scale implementation. The primary obstacles in scaling up nanoformulation production include (1) developing safe and effective nanoformulations through green nanotechnology approaches, (2) ensuring batch-to-batch reproducibility and controlled manufacturing processes, (3) achieving target-specific delivery while maintaining functionality, (4) guaranteeing long-term stability and structural properties, and (5) understanding nanoparticle exposure effects, including systemic toxicity, and characterizing in vivo behavior [25].

## 4. Polysaccharide-Based Nanocarriers for Plant-Derived Antimicrobials

Plant antimicrobials comprise a broad group of bioactive compounds demonstrating antioxidant, antibacterial, and antifungal activities [223]. Essential oils (EOs) and plant extracts (PEs) represent complex mixtures containing diverse phytochemicals, including monoterpenes, phenylpropanoids, monoterpenoids, phenolic compounds, and other major substance classes that serve as effective antimicrobial agents [4,35,224,225]. Notable examples of these plant-derived antimicrobials include curcumin, quercetin, saponin, resveratrol, gallic acid, magnoflorin, sulforaphane, naringenin, honokiol, glycyrrhetinic acid, rutin, luteolin, carotene, α-tocopherol, zoledronic acid, kaempferol, carvacrol, thymol, linalool, and menthol, among others—all exhibiting superior antimicrobial and antifungal properties [4,14,221,222].

Chitosan is extensively utilized in nanoparticle (NP) formulation (Table 3) due to its intrinsic antimicrobial properties [121,226,227]. The formulation of nanoparticles (NPs) with plant essential oils (EOs) typically requires emulsification [228], followed by biopolymer-specific techniques such as ionic gelation or coacervation for chitosan and alginate [229,230,231,232]. Chitosan-based NPs incorporating tea water extract effectively inhibited *P. grisea* and *X. oryzae* [233], while those with grape pomace extract demonstrated significant antimicrobial efficacy: a 6-log reduction in *C. albicans*, 5-log reduction in methicillin-susceptible *S. aureus* (MSSA), 3-log reduction in *L. monocytogenes* and *P. aeruginosa*, and 1-log reduction in *E. coli* and *S. enteritidis* [234]. Similarly, chitosan NPs containing *Lavandula angustifolia* water extract suppressed biofilm formation in *P. aeruginosa*, *S. aureus*, and *C. albicans* [235], and those with *Martynia annua* leaf ethanol extract showed strong antibacterial activity (in descending order): *B. fragilis* > *S. oralis* > *P. acnes* > *P. aeruginosa* > *S. aureus* > *E. coli* > *B. cereus* > *S. mutans* > *A. hydrophila* > *S. faecalis* [236]. Clove and guava leaf essential oils (EOs) were successfully incorporated into chitosan NPs through oil-in-water emulsification followed by ionic gelation. The resulting NPs exhibited significant antimicrobial activity against *L. monocytogenes*, *S. aureus*, *S. typhi*, *E. coli* [229], and *K. pneumoniae* [230]. Resveratrol, a potent polyphenolic antioxidant, demonstrates broad-spectrum antimicrobial properties against diverse bacteria, viruses, and fungi [237]. When encapsulated in chitosan NPs, resveratrol showed enhanced activity against *H. pylori* [238].

Chitosan is synergistically combined with alginate, pectin, or gums through electrostatic interactions to form complex nanostructures with enhanced stability and broad-spectrum antimicrobial activity [239,240,241,242]. These hybrid systems employ techniques like pre-ionic gelation (for chitosan/alginate or chitosan/pectin NPs) and complex coacervation [240,243,244], as demonstrated by oregano EO- and *Ocimum sanctum*-loaded chitosan/alginate NPs effective against *S. aureus* [243], *E. coli*, *P. aeruginosa*, and *B. cereus* [244]. Anthocyanins, a class of bioactive water-soluble flavonoid pigments [245], exhibit broad-spectrum antimicrobial activity with particularly pronounced efficacy against Gram-positive bacteria [246]. These compounds have been successfully encapsulated into hybrid chitosan/pectin NPs [241].

**Table 3:** Representative polysaccharide-based nanoformulations incorporating plant-derived antimicrobial compounds.

Active Component	Nanocarrier	Composition	Formulation Method	Antimicrobial Activity	Ref. and Pub. Year
Tea water extract (TPS)	Chitosan	Chitosan (85% DA, MW 27 kDa, 250 mg/100 mL) TPS (2.5 mg/mL)	Complex coacervation	Against *P. grisea*, *X. oryzae*	[233] 2024
*Lavendula angustifolia* water extract		Chitosan (1% *w*/*v*) Ratio to extract (1:1)	Self-assembling	Suppressed *P. aeruginosa*, *S. aureus*, and *C. albicans* biofilm formation	[235] 2023
*Martynia annua* leaves ethanol extract		Chitosan (1% *w*/*v*) Ratio to extract (1:1)		Considerable antibacterial activity in order of *B. fragilis* > *S. oralis* > *P. acnes* > *P. aeruginosa* > *S. aureus* > *E. coli* > *B. cereus* > *S. mutans* > *A. hydrophila* > *S. faecalis*	[236] 2022
Clove essential oil (CEO)		Chitosan (75–85% DA, MW 50–190 kDa, 1% *w*/*v*) Ratios to CEO (1:0, 1:0.25, 1:0.5, and 1:1) Sodium tripolyphosphate (TPP)	Oil-in-water emulsification followed by ionic gelation	Against *L. monocytogenes*, *S. aureus*, *S. typhi*, and *E. coli*	[229] 2020
Guava leaves essential oil (GLEO)		Chitosan (75% DA, MW 50 kDa, 1% *w*/*v*) Ratio to GLEO (1:1) TPP		Against *K. pneumoniae*	[230] 2020
Curcumin		Chitosan (1 mg/mL) Curcumin stock (1 mg/mL) in ethanol TPP (1 mg/mL)	Ionic gelation	Against *S. aureus* and *P. aeruginosa*	[247] 2014
Resveratrol		Chitosan (75–85% DA, MW 50–190 kDa, 2 mg/mL) Resveratrol stock (5 mg/mL) in ethanol Ratio (1:5) TPP (1 mg/mL)		Against *H. pylori*	[238] 2024
Grape pomace extract		Chitosan (low molecular weight) Grape pomace extract TPP (1 mg/mL)		6-log reduction in *C. albicans*, 5-log reduction in MSSA, a 3-log reduction in *L. monocytogenes* and *P. aeruginosa*, and a 1-log reduction in *E. coli* and *S. enteritidis*	[234] 2021
Grape pomace extract	Alginate	Sodium alginate Grape pomace extract Calcium chloride (2 mg/mL)	Ionic gelation	6-log reduction in *C. albicans*, 3-log reduction in MSSA, a 2-log reduction in *L. monocytogenes*, *P. aeruginosa*, and *S. enteritidis*, and 1-log reduction in *E. coli*	[234] 2021
*Cuminum cyminum* and *Zataria multiflora* EOs		Alginate solution (0.25% *w*/*v*) EO (0.25% *w*/*v*) Calcium chloride (0.04–0.05%)	Oil-in-water emulsification followed by ionic gelation	Superior efficacy for NPs containing *Z. multiflora* EO against *E.coli*, *P.aeruginosa*, and *S. aureus*	[231] 2024
Lemon EO		Alginate Myristic acid Ethylene carbo-di-imide hydrochloride (EDC) N-hydroxysuccinimide (NHS) Addition of EO in drop-wise manner	Emulsification and cross-linking	Inhibit multidrug-resistant (MDR) isolates of *Acinetobacter baumannii*	[232] 2025
Oregano EO	Chitosan Alginate	Chitosan (MW 110–150 kDa) Alginate (very low viscosity) Calcium chloride Oregano EO	Oil-in-water emulsification followed by pre-ionic gelation of alginate and coacervation with chitosan	Against *S. aureus*	[243] 2022
*O. sanctum* methanolic extract		Sodium alginate solution (0.06%, *w*/*v*) Calcium chloride (18 mM) Chitosan solution (0.05%, *w*/*v*) Methanolic extract of O. sanctum	Pre-ionic gelation of alginate followed by chitosan coacervation	Against *E. coli*, *P. aeruginosa*, *B. cereus*, and *S. aureus*	[244] 2013
Curcumin		Sodium alginate (3%) Chitosan (75–85% DA, low MW) Ratio (5:4) Curcumin dissolved in ethanol	Coacervation	Mild activity against *S. aureus*, *B. subtilis*, and *E. aerogenes*	[248] 2024
*Terminalia arjuna* (arjuna), *Azadirachta indica* (neem), *Withania somnifera* (ashwagandha), *Tinospora cordifolia* (giloy), *Murraya koenigii* (curry leaves) extracts	Bacterial Nanocellulose (BNC)	BNC Extracts (20% *w*/*v* in water)	Ex situ modification of BNC by simple dipping method	Against *E. coli* and *A. viridans*	[249] 2020
Curcumin		BNC Curcumin and curcumin degradation products (0.05, 0.1, and 0.5 mg/mL)	Loaded from aqueous solution during autoclaving	Against *S. epidermidis* and *E. coli*	[250] 2020
Cell cultures of *Chelidonium majus*		BNC *C. majus* cells	*C. majus* cells were cultured in vitro on BNC matrices in liquid media, followed by enzymatic digestion and purification	Against *S.aureus*, *P. aeruginosa*, and *C. albicans*	[251] 2022
Peppermint (PM), Cinnamon (CN) and lemongrass (LG) EOs	Cellulose Acetate (CA)	CA (acetyl content of 39.8%, MW 30 kDa, 1% *w*/*v*) EO in acetone (0.5% *v*/*v*)	Nanoprecipitation by solvent/anti-solvent technique EOs were grafted on cellulose acetate molecules	CA/CN significantly inhibited growth of *S. aureus*, *E. coli*, *P. aeruginosa*, and *C. albicans*	[252] 2018
Thymol	Cellulose Nanofibrils (CNFs)	CNFs Thymol (200 mg)	Impregnation with thymol in scCO_2_	Against *E. coli*, *S. epidermidis*, and *C. albicans*	[253] 2020
Curcumin	Xylan	Xylan (0.132 g, 1 mmol) Curc-monosuccinate (0.864 g, 2 mmol) DMSO N, N’-dicyclohexylcarbodiimide (DCC, 0.412 g, 2 mmol) 4-Dimethylaminopyridine (DMAP, 0.116 g, 1 mmol) Precipitation in ethanol/ethyl ether (1:1 *v*/*v*)	Conjugation followed by precipitation	No data	[254] 2018
Menthone, oregano, cinnamon, lavender, and citral EOs	Starch	Debranched starch (1% *w*/*v* in water) EOs (250 µL dissolved in 20 mL hot ethanol)	Precipitation and freeze drying	Better antimicrobial activity against *S. aureus* than *E. coli*	[255] 2017
*Triphala Churna* (TC) extract		Heating soluble starch (5 g) in 0.4 M NaOH Addition of 0.3% of TC and acetone	Precipitation and graft copolymerization	Antibacterial activity against *S. typhi and S. dysenteriae*; antibiofilm activity against ATCC MRSA 33591 and clinical strain N7	[256] 2020
Linalyl acetate		Corn starch (2 wt%) Mixture of 1 M sodium hydroxide and 1 M urea, volume ratio 1:2 Linalyl acetate (1.5 wt%) Tween 80 Ethanol was used as anti-solvent (1:15 ratio to solvent)	Nanoprecipitation by solvent/anti-solvent technique	Promote wound healing	[257] 2025
Curcumin		Cinnamic acid-esterified debranched starch Curcumin	Additional π-π interactions provided from cinnamic acid	Biofilm scavenging ability, superior antibacterial effects	[258] 2022
Quercetin		Pea, corn and potato starches (20 mg/mL) and quercetin (2 mg/mL) dissolved in NaOH/urea/H_2_O Ratio (0.8:1:98.2 by weight) 0.1 M HCl	Nanoprecipitation	No data	[259] 2018
Rutin		Quinoa and maize starch suspensions (1.5%) preheated (80 °C) in 0.1 M NaOH solution Rutin (1.5%) dissolved in ethanol Ratio (1:10)	Ultrasonication		[260] 2021
Flavonoids of citrus peel extracts (CPE)	Pectin	Pectin water solution CPE ethanol extract Calcium chloride	Ionic gelation	No data	[261] 2017
Quercetin	Pectin Chitosan	Chitosan (80% DA, MW 190–300 kDa, 1% *w*/*v*) TPP Quercetin dehydrate Pectin Calcium chloride			[240] 2022
Anthocyanins (ANCs)		Chitosan (95% DA, MW 300 kDa, 1% *w*/*v*) ANCs (1–4% *w*/*v*) Pectin (MD 30%, 5% *w*/*v*) Mass ratio of chitosan/pectin/anthocyanin (1:1:3)	Coacervation		[241] 2020
*Lippia sidoides* EO	Chitosan Cashew Gum	Gum (MW 110 kDa, 5%) Chitosan (75% DA, MW 180 kDa) Ratio (1:1) Polymer matrix/EO (10:2)	Complex coacervation	Against *St. Aegypti larvae*	[242] 2012
Saffron extract	Chitosan Arabic Gum	Chitosan (DA > 75%, MW 50–180 kDa, 1–10 mg/mL) Gum arabic (MW 295–1860 kDa, 1–5 mg/mL) Saffron (5–15 mg/mL)		No data	[262] 2019
*L. sidoide* EO	Alginic Acid Sodium Salt Cashew Gum	Alginate (low viscosity, MW 54 kDa) Cashew gum (MW 110 kDa) Relative ratios of alginate/gum (1:3, 1:1, and 3:1) Blend/oil ratio (10:1, *w*/*w*) Calcium chloride (0.5%, *w*/*w*)	Ionic gelation followed by spray drying		[263] 2014
Curcumin	*Prunus armeniaca* Gum (PAGE)	PAGE solution in water Ethanolic solution of curcumin (400 μL, 10 mg/mL) Calcium chloride-to-mixture ratio (1:1)	Ionic gelation	Against *S. aureus* and *E. coli*	[264] 2021
Quercetin		PAGE Quercetin Calcium chloride		Significant decline in bacterial load	[265] 2024
D-limonene (DL, R-(+)-Limonene)	Carrageenan	Carrageenan (sulfate content around 27% *w*/*w*, MW 672 ± 32 kDa) DL	Electrospray	No data	[266] 2022
Curcumin		Carrageenan (0.15% and 0.44% in 0.5 mL/L NaCl aqueous solution) Curcumin ethanolic solution (100 mg/mL and 10 mg/mL)	Self-assembling		[139] 2022
Grapefruit seed extract and cinnamon oil (GCN)	Chitosan Carrageenan	Chitosan Carrageenan GCN	Complex coacervation	Against *Streptococcus mutans* and *sobrinus*	[267] 2023
Quercetin	Modified Dextran	Grafted dextran with L-cysteine and octadecylamine onto carboxymethyl dextran Quercetin	Self-assembling	No data	[268] 2023
*Eucalyptus staigeriana* EO	Dextran Sulfate Chitosan	Dextran sulfate Chitosan Aloe Vera *Eucalyptus staigeriana* EO	Formation of hydrogel	Inhibited bacteria growth	[269] 2023
α-Tocopherol		Dextran sulfate (MW 15 kDa) Chitosan (95%DA) α-Tocopherol Lecithin	Multi-layer nanoemulsions	No data	[270] 2024
Curcumin		Dextran sulfate (MW > 500 kDa, 0.1 wt%) Chitosan (DA > 75%, low MW, 0.1 wt%) Volume ratio of 3:2 Curcumin (2 mg) was loaded into NPs in 5 wt% of polymer	Complex coacervation		[271] 2011
Naringenin		Dextran sulfate (MW 500 kDa, 0.1 wt%) Chitosan (DA > 75%, 0.1 wt%) Volume ratio (3:2) Naringenin (2 mg/mL) was equal to 5% weight of polymers			[272] 2021
Clove extract	Pullulan Whey	Pullulan (20% *w*/*w*) Whey (20% *w*/*w*) Pullulan: whey protein ratios (100:0 *w*/*w*, 50:50 *w*/*w*, and 25:75 *w*/*w*) Clove extract (5% *w*/*w*)	Electrospinning	Against *S. aureus* and *M. luteus*	[273] 2022
Resveratrol	Pullulan	Pullulan Resveratrol	Surface-functionalized with the ligand n-acetyl glucosamine	No data	[274] 2025
Tannic acid	Pullulan Chitosan	Pullulan (18 wt%) Chitosan (DA 75–85%, MW 50–190 kDa, 3 wt%) Tannic acid (1 wt%)	Force-spinning	Against *E.coli*	[275] 2015
Curcumin	Pullulan Hyaluronic Acid (HA)	Succinylated pullulan (SPu, 200 kDa, 400 mg) HA (5.4 kDa, 0.528 mmol disaccharide repeat unit) DMAP (0.106 mmol) EDC (0.528 mmol) Formamide Curcumin Mass ratios Cur/HA-SPu (1/5, 1/10, 1/15)	Conjugation	Against *E. coli* and *S. aureus*	[276] 2020
Olive leaf extract (OLE)	Hyaluronic Acid Silk Fibroin (SF)	SF (15% *w*/*v* in formic acid) HA (0.5% *w*/*v* in distilled water) OLE (12 and 15% *w*/*v*)	Electrospinning	Perfect antibacterial activities against both Gram-negative and Gram-positive bacteria, while antifungal activity against *C. albicans* was rather poor	[277] 2016
Curcumin	Pluronic Chitosan Hyaluronic Acid	Pluronic Chitosan (DA 97%, MW 1–3 kDa) HA Triethanolamine (TEA) DMAP	Conjugation and nebulisation	No data	[278] 2021
Curcumin (CUR) and resveratrol (REV)	Hyaluronic Acid Chitosan	Chitosan (0.1% *w*/*v*) 1 mg of each CUR and REV is dissolved into 70:30 ratio of ethanol and water TPP (0.1% *v*/*v*) HA (0.01–0.05% *w*/*v*)	Ionic gelation followed coacervation		[279] 2020
Quercetin	Hyaluronic Acid	HA sodium salt solution (0.5% *w*/*v*, MW 200 kDa) DMSO (1:1 *v*/*v* ratio) Glutaraldehyde (5% *v*/*v*) HCl Quercetin (34 µmol) in phosphate buffer containing 10% *v*/*v* of ethanol	Nanoprecipitation with solvent–non solvent method and cross-linking		[280,281] 2017 2018
Tannic acid (TA)	Sodium Hyaluronate (HA)	HA (MW 44 KDa (low), 375 kDa (medium) and 737 kDa (high), 10 mg/mL) 4-(4,6-dimethoxy-1,3,5-triazin-2-yl)-4-methylmorpholinium chloride (DMTMM, 404 μmol) 3-aminophenylboronic acid hydrochloride (3-APBA∙HCl, 91 μmol) HA-APBA (2 mg/mL) TA water solution (28, 56, 112, 224, or 448 μg/mL)	Cross-linking (Catechol/Boronate complexation)	Against *E. coli*, MSSA, and MRSA	[282] 2016

Notes: TPS—tea water extract; DA—deacetylation degree; MW—molecular weight; CEO—cove essential oil; GLEO—guava leaf essential oil; TPP—sodium tripolyphosphate; MSSA—methicillin-susceptible S. aureus; EDC—ethylene carbo-di-imide hydrochloride; NHS—N-hydroxysuccinimide; MDR—multidrug-resistant; EO—essential oil; BNC—bacterial nanocellulose; CA—cellulose acetate; PM—peppermint; CN—cinnamon; LG—lemongrass; CNFs—cellulose nanofibrils; DCC—N, N’-dicyclohexylcarbodiimide; DMAP—4-Dimethylaminopyridine; TC—Triphala Churna; MRSA—methicillin-resistant S. aureus; CPE—citrus peel extract; ANCs—anthocyanins; MD—methylation degree; PAGE—Prunus armeniaca gum exudate; DL—D-limonene; GCN—grapefruit seed extract and cinnamon oil; SPu—succinylated pullulan; HA—hyaluronic acid; OLE—olive leaf extract; SF—silk fibroin; TEA—triethanolamine; DMTMM—4-(4,6-dimethoxy-1,3,5-triazin-2-yl)-4-methylmorpholinium chloride; 3-APBA∙HCl—3-aminophenylboronic acid hydrochloride.

*Lippia sidoides* essential oil and saffron extract were effectively loaded into chitosan/gum NPs via complex coacervation [242,262], while chitosan/carrageenan NPs containing grapefruit seed extract and cinnamon oil demonstrated strong activity against *St. mutans* and *S. sobrinus* [267]. Alginate NPs with grape pomace extract exhibited dose-dependent antimicrobial effects, achieving a 6-log reduction in *C. albicans*; 3-log reduction in MSSA; 2-log reduction in *L. monocytogenes*, *P. aeruginosa*, and *S. enteritidis*; and 1-log reduction in *E. coli* [234]. Furthermore, alginate NPs encapsulating *Cuminum cyminum*, *Zataria multiflora*, or lemon essential oils (prepared by oil-in-water emulsification with ionic gelation/cross-linking) showed species-specific activity: *Z. multiflora*-loaded NPs were effective against *E. coli*, *P. aeruginosa*, and *S. aureus* [231], whereas lemon oil NPs targeted multidrug-resistant *A. baumannii* [232]. Additionally, citrus peel flavonoids were successfully incorporated into pectin NPs through ionic gelation [261], further expanding the repertoire of plant-polysaccharide antimicrobial delivery systems.

Curcumin and quercetin exhibit strong antimicrobial potential, though their therapeutic applications are limited by poor water solubility and low bioavailability [283,284]. These challenges have been addressed through advanced nanoformulations: (1) curcumin-loaded NPs using *Prunus armeniaca* gum, chitosan, and chitosan/alginate composites demonstrated efficacy against *S. aureus* [247,248,264], *E. coli* [264], *P. aeruginosa* [247], *B. subtilis*, and *E. aerogenes* [248]; (2) quercetin was successfully encapsulated in *Prunus armeniaca* gum and chitosan/pectin NPs via ionic gelation [240,265], as well as in hyaluronic acid nanostructures through nanoprecipitation with solvent–non-solvent methods and cross-linking [280,281]. Additional curcumin delivery systems include BNC loading during autoclaving, carrageenan encapsulation, and xylan conjugation [139,250,254]. Furthermore, formulations like succinylated pullulan/hyaluronic acid and pluronic/chitosan/hyaluronic acid NPs have enhanced curcumin’s activity against *E. coli* and *S. aureus* [276,278], demonstrating the versatility of polysaccharide-based nanocarriers for these bioactive compounds.

Nanocellulose possesses exceptional physicochemical properties, including high surface area, porosity, and modifiable surface chemistry, making it a suitable platform for antimicrobial agent encapsulation and immobilization [285]. BNC can incorporate natural antimicrobials or living cell cultures through various approaches: physical adsorption, in situ incorporation, immersion techniques, chemical fixation, or electrostatic self-assembly [251,286]. Hemicelluloses (xylans, mannans, and β-glucans) demonstrate synergistic effects with polyphenolic compounds and can be functionalized through either physical adsorption or covalent conjugation, while nanoparticles are typically prepared via precipitation or dialysis methods [127,287]. BNC composites incorporating traditional medicinal plant extracts (*Terminalia arjuna*, *Azadirachta indica*, *Withania somnifera*, *Tinospora cordifolia*, and *Murraya koenigii*) or *Chelidonium majus* cell cultures was prepared by dipping or cultivation with subsequent enzymatic digestion and exhibited broad-spectrum antimicrobial activity against *E. coli*, *A. viridans* [249], *S.aureus*, *P. aeruginosa*, and *C. albicans* [251]. Notably, cinnamon essential oil encapsulated in cellulose acetate nanoparticles shows potent inhibitory effects against clinically relevant pathogens including *S. aureus*, *E. coli*, *P. aeruginosa*, and *C. albicans* [252]. Thymol, a bioactive monoterpenoid phenol abundant in essential oils, demonstrates particularly strong antimicrobial properties [288] when impregnated into cellulose nanofibrils using supercritical CO_2_ technology, showing efficacy against *E. coli*, *S. epidermidis*, and *C. albicans* [253].

Starch NPs are typically prepared via precipitation [115] and effectively encapsulate diverse bioactive compounds including phytochemicals, essential oils (EOs), and plant extracts [255,256,257,258,259,260], often requiring debranching pretreatment for optimal formulation [255,258]. These NPs with EO-loaded systems (menthone, oregano, cinnamon, lavender, and citral) showed greater efficacy against *S. aureus* than *E. coli* [255]. *Triphala Churna* extract-loaded starch NPs exhibit dual activity: antibacterial action against *S. typhi* and *S. dysenteriae*, plus antibiofilm effects against methicillin-resistant *S. aureus* (MRSA) ATCC 33591 and clinical strain N7 [256]. The platform also successfully encapsulates linalyl acetate, curcumin, quercetin, and rutin [257,258,259,260].

Dextran derivatives and their NPs demonstrate antimicrobial properties [289,290]. Quercetin has been successfully encapsulated into self-assembled NPs prepared from dextran grafted with L-cysteine and octadecylamine [268]. Dextran sulfate and chitosan were combined to incorporate *Eucalyptus staigeriana* essential oil (EO), α-tocopherol, curcumin, and naringenin through three fabrication methods: complex coacervation [271,272], multilayer nanoemulsions [270], and antibacterial hydrogels [269]. Resveratrol, a bioactive polyphenol with demonstrated antibacterial and antibiofilm properties [291], has been effectively encapsulated in both pullulan-based structures [274] and hybrid hyaluronic acid/chitosan systems via ionic gelation–coacervation, with the latter showing synergistic effects when combined with curcumin [279].

Equipment-based spinning techniques were employed to fabricate nanoformulations of pullulan/whey, pullulan/chitosan, and hyaluronic acid/silk fibroin. These systems incorporated clove extract, olive leaf extract, and tannic acid, demonstrating antimicrobial activity against *S. aureus* and *M. luteus* [273], *E. coli* [275,277], and MRSA [277]. Tannic acid, a natural polyphenolic tannin, exhibits broad-spectrum antimicrobial and antiviral properties [292]. Sodium hyaluronate NPs containing tannic acid showed significant efficacy against *E. coli*, MSSA, and MRSA [282].

Plants represent a rich source of diverse secondary metabolites, such as tannins, terpenoids, alkaloids, and flavonoids, which exhibit broad-spectrum bioactive properties, particularly antimicrobial activity [293]. These phytochemicals can be effectively encapsulated within polysaccharide nanomatrices in various forms (essential oils, crude extracts, or purified compounds), serving as potent antimicrobial agents with sustained release profiles [294,295].

## 5. Polysaccharide-Based Nanocarriers for Microbial-Derived Antimicrobials

Microorganisms produce diverse antimicrobial agents, including peptides, spirotetronates, polyketides, alkaloids, organic acids, and sesquiterpene derivatives [296]. Among these, bacteriocins represent one of the most promising and intensively studied groups of microbial-derived antimicrobials [4,297,298,299,300]. Bacteriocins are classified into three main categories based on their chemical structure, molecular weight, biochemical properties, spectrum of antimicrobial activity, and mechanism of antimicrobial action: (1) Class I—heat-stable lanthionine-containing peptides (lantibiotics, thiopeptides, sactibiotics, lasso peptides, and cyclic bacteriocins); (2) Class II—small heat-stable non-lanthionine peptides; and (3) Class III—large heat-labile proteins [224,301,302,303]. Class I bacteriocins include lantibiotics, lipolantins, thiopeptides, botromycins, linear azole-containing peptides, sactibiotics (sactipeptides), lasso peptides, and cyclic bacteriocins [303]. Class II bacteriocins are divided into three subclasses and include pediocin, enterocin, sakacins, leucocin, carnobacteriocins, etc. [304]. Class III bacteriocins include megacins, klebicin, helveticin I, and enterolysin [305].

While bacteriocins show promise as antimicrobial agents, they face several application challenges that limit their clinical and industrial utilization. Thus, bacteriocins have a limited spectrum of activity against closely related bacterial strains [306,307]. The intensive application of bacteriocins could lead to the potential development of bacterial resistance. Thus, some species of *B. cereus* and *P. polymyxa* produce nisinase, while *Listeria* species demonstrate alterations in the surface charge of their cell walls due to gene mutations that disrupt bacteriocin binding, and some *C. botulinum* strains also show cross-resistance to Class II bacteriocins [308]. Low production yield, purification challenges, and formulation stability issues hinder the widespread adoption of bacteriocins as biopreservatives [309,310]. Due to their peptide nature, bacteriocins are sensitive to proteases, possess poor pharmacokinetic profiles, and may act as host sensitizers or allergens [311]. These limitations may be partially overcome through nanoencapsulation strategies.

Nisin is a widely recognized Class I bacteriocin with Generally Recognized as Safe (GRAS) status [312,313]. Composed of 34 amino acids, it is produced by *Lactococcus lactis* ssp. [314] and exhibits potent inhibitory activity against spore-forming bacteria such as *Bacillus* and *Clostridium*. Additionally, nisin demonstrates efficacy against *Listeria*, *Micrococcus*, *Staphylococcus*, *Streptococcus*, *Lactobacillus*, *Lactococcus*, *Leuconostoc*, *Mycobacterium*, and *Pediococcus*. However, it shows minimal to no activity against Gram-negative bacteria [315]. Nisin-incorporated nanoparticles (NPs) were successfully developed using various biopolymers, each demonstrating distinct antimicrobial properties. Nisin was encapsulated into chitosan NPs via ionic gelation (Table 4), exhibiting antimicrobial activity against *S. aureus*, *L. monocytogenes*, and *E. coli* [316,317]. Additionally, nisin-loaded NPs were fabricated using chitosan combined with alginate [318,319], pectin [320], or carrageenan [321] through electrostatic coacervation. These NPs effectively suppressed the growth of *L. monocytogenes*, *S. aureus, E. coli*, *S. enterica*, *M. luteus*, *P. aeruginosa*, and *E. aerogenes*. Bacterial cellulose nanocrystals, carboxymethylcellulose, and nanofibrillated cellulose can form nanocomposites with nisin through complexation. These nanocomposites exhibit microbial inactivation and demonstrate activity against *S. aureus* and *B. subtilis* [322,323,324]. Co-culturing *Enterobacter* sp. FY-07 (a bacterial nanocellulose producer) with *Lactococcus lactis* N8 (a nisin producer) resulted in the formation of a nanomaterial with strong inhibitory effects against Gram-positive bacteria [325]. Nisin-functionalized cellulose nanofibers exhibit inhibitory activity against *B. thermosphacta* and *L. innocua* [326], while holocellulose nanofibrils conjugated with nisin demonstrate antimicrobial efficacy, particularly against Gram-positive bacteria including *S. aureus* and *L. monocytogenes* [327].

Alginate–nisin and alginate–starch–nisin NPs, prepared by emulsification followed by ionic gelation showed effective activity against *L.monocytogenes* [328]. Nisin-incorporated pectin NPs exhibited spectrum-specific antimicrobial activity that varied with the esterification degree, showing efficacy against *Arthrobacter* sp., *B. subtilis*, *E. coli*, and *Klebsiella* sp. [329]. Notably, high-methoxyl pectin nanoparticles demonstrated inhibition against *S. aureus* and *E. coli* [330]. Similarly, gellan gum- and dextran-based nisin nanoparticles showed potent activity against S. aureus [295]. Similarly, gellan gum- and dextran-based NPs with nisin were active against *S. aureus* [331], and radiation-synthesized dextran-nisin conjugates showed broader spectrum activity against *E. coli*, *P. fluorescence*, *S. aureus*, and *B. cereus* [332]. Electrospun NPs fabricated from pullulan, amaranth protein isolate, and nisin demonstrated antimicrobial effects against *L. mesenteroides*, *L. monocytogenes*, and *S. Typhimurium* [333]. Hyaluronic acid-based formulations showed promising results: HA–nisin nanoformulations prepared by electrostatic complexation exhibited superior inhibition against hyaluronidase-producing *S. aureus* compared to *B. cereus* [334], while HA–nisin conjugates were also effective against *S. epidermidis*, *S. aureus*, and *P. aeruginosa* [335].

Pediocin-like bacteriocins are small (<5 kDa) peptides characterized by the conserved sequence -Y-G-N-G-V-X_1_-C-X_2_-K/N-X_3_-X_4_-C- [349], produced primarily by some *Pediococcus* spp. [350]. They exhibit broad-spectrum activity against Gram-positive bacteria, with particularly strong inhibition of *L. monocytogenes*, as well as efficacy against *E. faecalis*, *S. aureus*, and *C. perfringens* [350,351,352]. When encapsulated in alginate–guar gum via complex coacervation, pediocin demonstrated significant activity against *L. innocua* [336]. Plantaricins, bacteriocins derived from *L. plantarum* [353], include variants such as plantaricin E/F, which inhibit Gram-positive bacteria. Alginate-encapsulated plantaricin E/F showed antimicrobial effects against the sensitive indicator strain *L. plantarum* NCIMB 700965 (LP965) [337]. Enterocins, produced by *Enterococcus* spp. [354,355], display potent activity against foodborne pathogens, including *S. aureus*, *L. monocytogenes*, and *S.* enterica et al. [356]. Alginate and bacterial cellulose nanomaterials were successfully fabricated through simple ball milling [338] or soaking methods [339], demonstrating effective antimicrobial activity against *C. perfringens* and *L. monocytogenes*. In parallel, sakacins—a group of bacteriocins produced by specific *L. sakei* strains with a narrow antibacterial spectrum [357]—showed activity when conjugated with bacterial cellulose nanocrystals, particularly against *L. innocua* [340]. Further developments in bacteriocin delivery systems include chitosan nanoparticles produced by ionic gelation encapsulation of *Levilactobacillus brevis* and *Lactococcus lactis* subsp *lactis* bacteriocins. These nanoparticles exhibited superior antibacterial effects against Gram-positive pathogens (especially under acidic conditions) compared to Gram-negative bacteria [341] and demonstrated activity against *S. typhimurium*, *E. coli*, *B. cereus*, and *S. aureus* [342]. Similarly, cellulose nanocrystals functionalized with bacteriocins from *P. acidilactici* and *E. faecium* effectively inhibited the growth of multiple pathogens including *S. aureus*, *L. monocytogenes*, *E. coli*, *E. herbicola*, *B. subtilis*, *B. cereus*, and *P. aeruginosa* [343].

Natamycin, a natural antifungal compound produced by *Streptomyces* species, is widely approved as a food preservative [358,359]. While ineffective against bacteria, it demonstrates broad-spectrum activity against fungi and yeasts including *Candida* spp., *Aspergillus* spp., *Cephalosporium* spp., *Fusarium* spp., and *Penicillium* spp. [360]. Various nanoformulations have been developed to enhance its efficacy: chitosan-based nanoparticles (either alone or combined with zein or lecithin) showed strong activity against *C. albicans* [347], completely inhibiting spore germination and suppressing mycelial growth by 64.4% [344], with additional activity against *A. fumigates* [345]. Similarly, carboxymethylcellulose-gliadin NPs inhibited *P. expansum* [346], and alginate nanoparticles prepared by emulsification–ion gelation achieved a 2-log reduction in *A. flavus* populations [348].

Bacteriocins exhibit low toxicity to eukaryotic cells and demonstrate minimal inhibitory concentrations against numerous bacterial strains, along with high-temperature stability. However, they are sensitive to proteases, possess poor pharmacokinetic profiles, and may act as host sensitizers or allergens [311]. These limitations can be partially mitigated through nanoencapsulation, which enhances their stability and antimicrobial functionality [361].

## 6. Polysaccharide-Based Nanocarriers for Animal-Derived Antimicrobial Proteins and Peptides

Animal-derived antimicrobials include enzymes (e.g., lysozyme and lactoperoxidase), glycoproteins (lactoferrin, ovotransferrin, and avidin), histones, and antimicrobial peptides (arenicins, magainins, seroins, pleurocidins, cecropins, cathelicidins, protegrins, and defensins) [4,224,303,362].

Despite their potential as antimicrobial agents, animal-derived peptides and proteins face significant translational challenges that restrict their widespread clinical and industrial application, including a lack of selectivity, off-target effects, proteolytic instability, potential toxicity, and immunogenicity [363]. Low production yields and purification difficulties could also limit the application of animal-derived antimicrobial peptides and proteins [364]. Thus, studies have demonstrated that bacteria can develop resistance to AMPs under in vitro conditions [365]. Furthermore, certain antimicrobial peptides (AMPs) exhibit low specificity, targeting both pathogenic microbes and host cells. This non-selective activity can induce cytotoxic effects in human cells, resulting in adverse side effects that limit their therapeutic application [363,366]. Their proteinaceous nature renders animal-derived peptides and proteins susceptible to proteolytic degradation and results in suboptimal pharmacokinetic properties [367]. These limitations can be partially mitigated through nanoencapsulation.

Lysozyme, a 14.3 kDa secretory enzyme composed of 129 amino acids [368,369,370], is most abundant in egg white but also present in milk, cauliflower, cabbage, papaya juice, spleen, thymus, pancreas, and mucus [370], or it can be produced recombinantly [371]. Its antimicrobial activity primarily targets Gram-positive bacteria through the cleavage of β-(1,4)-glycosidic bonds in peptidoglycan, while it exhibits limited or negligible effects against Gram-negative bacteria [370,372]. Chitosan-based NPs incorporating lysozyme demonstrate broad-spectrum antimicrobial activity against both Gram-positive and Gram-negative bacteria through the synergistic action of chitosan and lysozyme (Table 5). Ionic gelation-fabricated chitosan-lysozyme NPs effectively inhibited the growth of *E. coli* and *B. subtilis* [373], while nanoprecipitated formulations significantly reduced *A. parasiticus* viability and strongly suppressed spore germination [374]. Lysozyme-conjugated chitosan NPs showed potent activity against *S. aureus*, *E. coli*, *P. aeruginosa*, and *K. pneumoniae* [375]. Nanogels fabricated from carboxymethyl chitosan, lysozyme, and amorphous calcium phosphate demonstrated activity against *S. mutans* [376]. Researchers have developed various innovative delivery systems for lysozyme, including chitosan/alginate NPs prepared via alginate pre-ionic gelation followed by chitosan coacervation [377], and complexes combining chitosan, CNCs, and lysozyme that exhibited antimicrobial effects against *E. coli* and *L. innocua* [378]. Additionally, lysozyme has been successfully encapsulated in depolymerized chitosan/dextran sulfate NPs through polyelectrolyte complexation [379] and in alginate formulations via ionic gelation [380], further expanding its potential applications in antimicrobial therapies. CNCs enable both nonspecific and covalent immobilization of lysozyme. The resulting nanomaterials exhibited antimicrobial activity against *M. deykticus* [381], *Corynebacterium* sp., *E. coli*, and *Ps. mendocina* [382].

In a separate formulation, cellulose acetate (CA) nanofibers were functionalized with pectin and lysozyme via electrostatic layer-by-layer assembly, yielding a nanomaterial with inhibitory effects against *E. coli* and *S. aureus* [383]. Starch-based formulations incorporating lysozyme have been successfully developed through cross-linking techniques [384,385], demonstrating potent antimicrobial activity against various bacterial strains including *B. licheniformis* 7558, *B. licheniformis* 6993, *B. subtilis* 168, *L. monocytogenes* LR991, and *L. monocytogenes* 001 [384]. Lysozyme-loaded NPs of pectin, κ-carrageenan, and xanthan gum were prepared through ionic gelation (retaining activity against *M. lysodeikticus*) [386] and complex coacervation via electrostatic polysaccharide–protein interactions [387,389,390], followed by either alkaline gelatinization [391,392] or high-pressure homogenization-assisted electrostatic complexation [393]. Gum arabic NPs incorporating lysozyme were fabricated through complex coacervation via electrostatic interactions, followed by Maillard reaction-induced conjugation, demonstrating antimicrobial activity against *E. coli* and *S. aureus* [388]. Similarly, lysozyme–dextran and lysozyme–pullulan conjugates prepared via Maillard dry heat processing [394,395,396,397] exhibited broad-spectrum inhibition against multiple pathogens, including *M. Lysodeikticus*, *V. parahaemolyticus* IFO 13286, *E. coli* IFO 12713, *A. hydrophila* IFO 13286, *P. mirabilis* IFO 12668, *K. pneumoniae* IFO 14438, *B. cereus* IFO 13690, *S. aureus* IFO 14462 [395], *E. coli*, *S. enterica*, and *S. aureus* [396]. Electrospun pullulan fibers cross-linked with lysozyme also showed efficacy against *E. coli* and *S. aureus* [397]. Additionally, hyaluronic acid–lysozyme complex coacervates [398,399] were developed with demonstrated wound-healing properties [399].

Lactoperoxidase (LPO) is an 80 kDa calcium- and iron-containing enzyme [432] predominantly found in mammalian secretions, particularly milk, where it constitutes ~1% (*w*/*w*) of whey proteins [433,434]. The LPO antimicrobial system, composed of lactoperoxidase (LPO), thiocyanate, and hydrogen peroxide, is naturally occurring and exhibits both bacteriostatic and bactericidal activity against diverse Gram-positive and Gram-negative microorganisms [435]. Lactoferrin (LF), a cationic glycosylated protein [436], is similarly abundant in milk (~1% of whey proteins) and colostrum, and it is also present in tears, saliva, gastric mucosa, spleen, lymph nodes, skin, and white blood cells [437]. While its primary role is iron binding, LF demonstrates broad-spectrum antibacterial activity [438], with reported efficacy against *E. coli*, *S. typhi*, *Streptococcus*, *L. pneumophila*, and *S. aureus* [439]. The LPO can be incorporated into NPs through complex coacervation, either with chitosan and gum tragacanth or in combination with LF, chitosan, and dextran [400,401,402,403]. Chitosan–LF NPs prepared by ionic gelation demonstrated antimicrobial activity against *S. aureus* [404]. Similarly, LF was successfully encapsulated in alginate formulations using ionic gelation [407,408], while pectin–LF NPs fabricated through complex coacervation [411,412] inhibited *P. aeruginosa* growth [411]. LF has also been conjugated with hyaluronic acid (either non-covalently or covalently) [413] or BNC, exhibiting antimicrobial effects against *S. aureus* and *E. coli* [409]. Additionally, the electrostatic complexation of LF with gellan gum formed NPs active against both *S. aureus* and *E. coli* [410]. Ovotransferrin (OVT), a 76 kDa glycoprotein constituting approximately 12% of total egg white protein [440], exhibits broad-spectrum antimicrobial activity against pathogens including *S. aureus*, *B. cereus*, *L. monocytogenes*, *E. coli*, and *H. pylori* [441]. Research has demonstrated OVT’s ability to form nanoformulations through polysaccharide–protein complexation with carboxymethyl chitosan, pectin, and various gums [414,415,416,417,418].

Antimicrobial peptides (AMPs), conserved across nearly all species, serve as components of innate host defense systems [362]. The Antimicrobial Peptide Database (APD) catalogs 2580 animal-derived AMPs, including 154 human host defense peptides, 397 from mammals, and 1110 active peptides from amphibians [442]. Their activity is largely attributed to cationic and amphipathic structural features [443], which enable broad-spectrum antimicrobial effects [444] through cell membrane disruption, the inhibition of cell wall synthesis, and interference with nucleic acid and protein production [445]. Chitosan is widely employed for encapsulating AMPs through ionic gelation, leveraging both the technique’s simplicity and chitosan’s inherent antimicrobial properties. This approach was proven successful with various AMPs. Cryptdin-2 from Paneth cells, when encapsulated in chitosan nanoparticles, reduced *Salmonella Typhimurium* loads in murine tissues by 2 log units [419]. Similarly, chitosan NPs incorporating frog skin-derived temporin B demonstrated efficacy against *S. epidermidis* [420], while those containing insect-derived cecropin-B showed activity against multidrug-resistant *K. pneumoniae* [421]. The encapsulation of human neutrophil defensin (HNP-1) in chitosan yielded NPs with broad-spectrum antibacterial activity against *S. aureus* ATCC 25923, *E. coli* NCTC 9001, *P. aeruginosa* ATCC 10145, *K. aerogenes* NCTC 10006, and MRSA [422]. Human cathelicidin peptide LL-37 encapsulated in chitosan NPs exhibited potent effects against MRSA [423]. Pleurocidin-like AMPs, identified across multiple flounder species, include NRC-07, which was successfully complexed with chitosan to form NPs exhibiting antimicrobial activity against *P. aeruginosa* [424].

AMPs have been successfully conjugated and immobilized into CNFs and modified starch matrices. These fabricated materials, incorporating either cecropin CA(1–7)M(2–9) or the LL-37 antimicrobial motif (KR-12), demonstrated growth inhibition against *B. subtilis* [425], *E. coli*, *S. aureus* [426,428], and even MRSA [428]. Electrospun pullulan fibers functionalized with the minimal bovine lactoferricin motif (LfcinB) (20–25)_Pal_ showed potent activity against *E. coli* [427]. Microfluidic chip technology enabled the fabrication of nanogels from octenyl succinic anhydride-modified hyaluronic acid conjugated with snake cathelicidin Ab-Cath. These nanogels demonstrated antimicrobial activity, inhibiting the growth *S. aureus*, *A. baumannii*, and *E. coli* in biological fluids while significantly reducing *S. aureus* and *A. baumannii* biofilms [429]. Cecropin B exhibited electrostatic interactions with hyaluronic acid in aqueous solutions [431]. Hyaluronic acid/PLGA complex coacervates incorporating insect thanatin from *Podisus maculiventri* thanatin [446] effectively mitigated sepsis caused by metallo-β-lactamases-1 (NDM-1) producing *E. coli* [430].

Synthetic AMPs are designed based on either known AMP structures or chemical composition–structure–activity relationships to achieve desired biological properties [447,448]. These engineered peptides address the key limitations of natural AMPs, including toxicity concerns, their short half-life, and their restricted antibacterial efficacy [449]. Synthetic AMPs have been successfully incorporated into various nanomaterial systems, including hyaluronic acid-based carriers [450], chitosan/alginate composites [451], alginate matrices [452], BNC platforms [453], and dextran formulations [454].

## 7. Conclusions

Natural antimicrobial compounds derived from plants, animals, and bacteria demonstrate significant potential while facing key application challenges, particularly their susceptibility to rapid degradation, poor pharmacokinetic profiles, short biological half-lives, potential allergenicity or toxicity, volatility, and hydrophobicity. Nanoencapsulation presents a promising solution to these challenges by improving compound stability, extending the circulation time, and enhancing antimicrobial functionality while minimizing adverse effects. Polysaccharides are renewable, widely accessible, biodegradable, and biocompatible, which makes polysaccharide-based NPs less toxic and allergenic. They are commonly used in polymer-based nanoformulations due to their excellent mechanical and physicochemical properties and their inherent biological activities, which can be further enhanced in nanoform.

Due to their advantageous properties, polysaccharide-based nanocarriers loaded with natural antimicrobials demonstrate high potential for applications in the food, cosmetic, and medical industries. In the food industry, these systems may be integrated both into packaging materials and directly into food products. A particularly promising application is their use for targeted delivery to specific regions of the gastrointestinal tract owing to the well-documented ability of certain polysaccharides to be selectively degraded in these regions by local enzymatic activity or microbiota. In addition, tissue engineering represents a highly relevant field for such systems as many polysaccharides inherently possess antimicrobial and/or regenerative activities. When combined with natural antimicrobial agents, these properties may result in a synergistic effect, enhancing the overall therapeutic efficacy of the nanocarriers.

However, the main challenges in using polysaccharides for nanoformulations include their variable chemical structure, which affects their physical and chemical properties; water absorption capacity; and poorly understood cytotoxic effects of their nano-forms. Moreover, challenges in process parameter optimization for their fabrication represent major barriers to industrial-scale implementation, as well as safety concerns. In addition, the issue of antimicrobial resistance remains a critical concern. The remarkable adaptability of microorganisms may limit the long-term effectiveness of such systems. Therefore, any practical implementation must undergo rigorous scientific and regulatory evaluation and will likely face usage restrictions to mitigate the risk of resistance development.

## Figures and Tables

**Table 1:** Chemical structures of polysaccharides.

Polysaccharide	Chemical Structure
Chitin	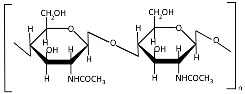
Chitosan	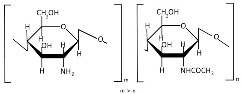
Alginate	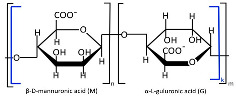
Cellulose	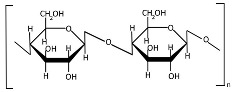
Representative hemicelluloses	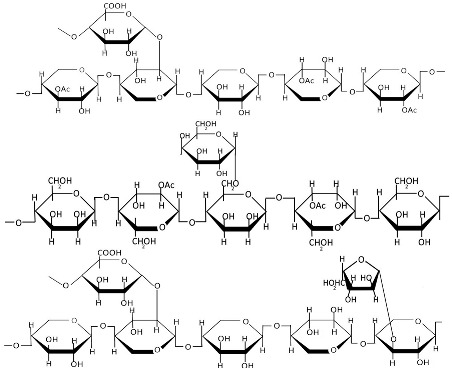
Starch	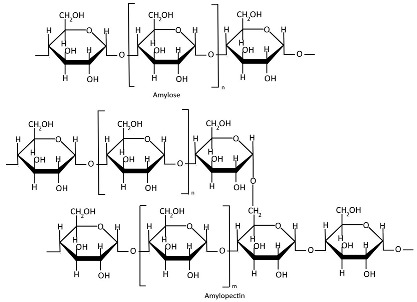
Homogalacturonan showing examples of methylation, acetylation, and amidation	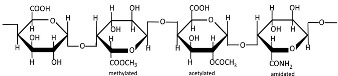
Carrageenan	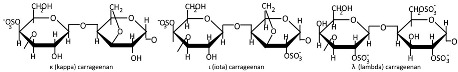
Dextran showing α-(1→6)-linked glucose backbone with potential α-(1→2), α-(1→3), or α-(1→4) branch points	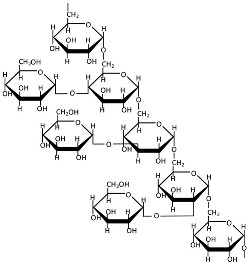
Pullulan	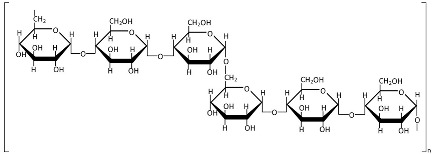
Hyaluronic acid	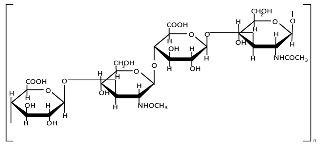

**Table 4:** Polysaccharide-based nanostructures incorporating microbial-derived antimicrobial agents.

Active Component	Nanocarrier	Composition	Formulation Method	Antimicrobial Activity	Ref. and Pub. Year
Nisin	Chitosan	Chitosan (MW 52 kDa, 0.5%) TPP (0.5%) Nisin (990 IU/mL) Nisin/TPP solution/chitosan ratio (1:10) D-trehalose (3% *w*/*v*)	Ionic gelation	Against *S. aureus* and *L. monocytogenes*	[316] 2018
		Chitosan (DA 90%, MW 10 kDa, 1% *w*/*v*) Nisin powder (2.5% *w*/*w* pure nisin, approximately 1,000,000 IU/g) TPP (0.5 mg/mL)		Against *E. coli* and *S. aureus*	[317] 2014
	Chitosan Alginate	Chitosan (75–85%, MW 50–190 kDa, 1% *v*/*v*) Alginate (5 mg/mL) Nisaplin (commercial form of nisin with 2.5% pure nisin of 1 × 10^6^ IU/g)	Alginate pre-ionic gelation followed by chitosan coacervation	Against *L. monocytogenes*	[318] 2018
		Chitosan (LMW, 250 mg/mL) Alginate (250 mg/mL) Nisin (450 IU/mL)		Against *S. aureus* and *L. monocytogenes*	[319] 2013
	Chitosan Pectin	Chitosan Pectin Nisin A (900 IU/mg)	Complex coacervation	Against *E. coli* and *S. enterica*	[320] 2025
	Chitosan Carrageenan	Chitosan Carageenan Tween 40 Nisin (900 IU/mg)		Against *M. luteus*, *P. aeruginosa*, *S. enterica*, and *E. aerogenes*	[321] 2014
	BNC	BCNs (5 mg/mL) Nisin (2.0 and 2.5 mg/mL)	Complexation	Microbial inactivation	[322] 2021
		Co-culturing *Enterobacter* sp. FY-07 (BNC) and *Lactococcus lactis* N8 (nisin)	Co-culture fermentation	Exhibits strong inhibitory activity against Gram-positive bacteria	[325] 2021
	CMC	Sodium CMC (MW 250 kDa, degree of substitution (DS) of 0.7, 0.9 and 1.2) Nisin (≥38,000 IU/mg)	Complexation	Against *S. aureus*	[323] 2023
	CNF	CNF (1.0—3.0% *w*/*v*) Nisin (640 AU/mL and 1280 AU/mL)	Simple mixing	Against *B. thermosphacta* and *L. innocua*	[326] 2022
	Nanofibrillated Cellulose	2,2,6,6-tetramethyl-1-piperidinyloxyl-oxidized nanofibrillated cellulose (TONFC, 0.75 mg/mL) Nisin (0.310 mg/mL)	Electrostatic complexation	Against *B. subtilis* and *S. aureus*	[324] 2018
Nisin	Holocellulose Nanofibrils (HCNF)	HCNF (3.5 g in 650 mL of deionized water) and NaIO_4_ (14 g) to form dialdehyde HCNF (DAC) 0.1 g of DAC and 25 mL of 0.35 g (0.2 M) hydroxylamine hydrochloride DAC (1 g) and nisin (1.6 g, 2.5%)	Conjugation	Antimicrobial effectiveness, particularly against Gram-positive bacteria such as *S. aureus* and *L. monocytogenes*	[327] 2025
	Alginate	Nisin Z^®^ (2.5% pure nisin) Sodium alginate (MW 197 kDa, mannuronate/guluronate ratio 0.6, 1% *w*/*v*) Sunflower oil with span 80 (1% *v*/*v*) and Tween 80 (1% *w*/*v*) Nisin/alginate weight ratio (4:1, 2:1 and 1:1) Calcium chloride solution (25% *w*/*w*)	Emulsification followed by ionic gelation	Against *L. monocytogenes*	[328] 2014
	Alginate Starch	Nisin Z^®^ (2.5% pure nisin) Sodium alginate (MW 197 kDa, mannuronate/guluronate ratio = 0.6, 1% *w*/*v*) Hi-maize^®^ 260 resistant starch (2 g to 100 mL nisin-alginate solutions) Sunflower oil with span 80 (1% *v*/*v*) and Tween 80 (1% *w*/*v*) Nisin/alginate weight ratio (4:1, 2:1 and 1:1) Calcium chloride solution (25% *w*/*w*)			
	Pectin	High-methoxyl pectin (HMP, MW 30–100 kDa, DE 60%) Low-methoxyl pectin (LMP, DE 26%) Pectin (0.4 mg/mL) Nisin (0.1—1 mg/mL)	Electrostatic complexation	Demonstrated antimicrobial activity against *Arthrobacter* sp., *B. subtilis*, *E. coli*, and *Klebsiella* sp., with efficacy dependent on biopolymer type	[329] 2016
		High methoxy pectin oligosaccharide (HMPOS, DE 85%) Nisin (4000 IU/mg) Mass ratio (HMPOS/nisin, 4:6)	Electrostatic complexation	Inhibitory effect on *S. aureus* and *E. coli*	[330] 2023
	Gellan Gum	Gellan gum (low acyl grade) Eudragit L100 in acetone Nisin/polymer/Eudragit L100 ratio (1:2:2)	Solvent evaporation	Against *S. aureus*	[331] 2024
	Dextran	Dextran 70 Eudragit L100 in acetone Nisin:polymer/Eudragit L100 ratio (1:2:2)			
		Dextran (MW 60–90 kDa) Nisin Nisin/dextran powder ratio (1:5 *w*/*w*)	Conjugation by irradiation	Against *E. coli*, *P. fluorescence*, *S. aureus*, and *B. cereus*	[332] 2012
	Pullulan Amaranth Protein Isolate	Pullulan Amaranth protein isolate Nisin	Electrospinning	Against *L. mesenteroides*, *L. monocytogenes*, and *S. Typhimurium*	[333] 2019
	Hyaluronic Acid (HA)	HA (0.4 mg/mL, 1 mM in anionic residues) Nisin (1.7 mg·mL^−1^, 3.0 mM in cationic residues 3:1 [N]/[COOH] ratio HEPES	Electrostatic complexation	Demonstrated superior inhibitory activity against hyaluronidase-producing *S. aureus* compared to *B. cereus*	[334] 2024
		HA (MW 1000 kDa, 2 mg/mL) NHS and 1-(3-dimethylaminopropyl)-N0 -ethyl-carbodiimide hydrochloride (EDC) EDC/NHS molar ratio of 1/1 for 1 eq of nisin Nisin (0.001 eq—0.01 eq for one carboxylic acid group of HA)	Conjugation	Against *S. epidermidis*, *S. aureus*, and *P. aeruginosa*	[335] 2014
Pediocin	Alginate Guar Gum	Alginate–guar gum solution (2% alginate plus 0.4% guar gum) Pediocin (20% to polymer solution)	Complex coacervation	Against *L. innocua*	[336] 2013
Plantaricin	Alginate	Sodium alginate (2% *w*/*w*) Calcium phosphate dibasic salt (0.2% *w*/*w*) Succinic acid (1% *w*/*w*) Plantaricin (0.0004 g)	Ionic gelation	Against sensitive indicator strain *L. plantarum* NCIMB 700965 (LP965)	[337] 2024
Enterocin	Alginate	Sodium alginate Enterocin 14 (EntDD14)	Ball milling method	Against *C. perfringens*	[338] 2021
	Bacterial Cellulose	Bacterial cellulose Cell-free supernatant (CFS) of *Enterococcus faecium* TJUQ1 Soak of BC in 80 AU/mL CFS for 6 h	Soaking	Against *L. monocytogenes*	[339] 2021
Sakacin	Bacterial Cellulose	Bacterial cellulose nanocrystals Sakacin-A	Electrostatic conjugation	Against *L. innocua*	[340] 2020
Bacteriocin of *Levilactobacillus brevis*	Chitosan	Chitosan (0.2% *w*/*v*) TPP Bacteriocin of *Levilactobacillus brevis*	Ionic gelation	Demonstrated superior antibacterial activity against Gram-positive pathogens, particularly under acidic conditions, compared to Gram-negative bacteria	[341] 2024
Bacteriocin from *Lactococcus lactis* subsp *lactis*		Chitosan (0.2% *w*/*v*) TPP Bacteriocin of *Lactococcus lactis* subsp *lactis*		Against *S. typhimurium*, *E. coli*, *B. cereus* and *S. aureus*	[342] 2021
Bacteriocins from *P. acidilactici* and *E. faecium*	Cellulose	Bacteriocin (0.2, 0.4, 0.6, 0.8, and 1.0 mg) Cellulose nanocrystals (8%)	Immobilization	Against *S. aureus*, *L. monocytogenes*, *E. coli*, *E. herbicola*, *B. subtilis*, *B. cereus*, and *P. aeruginosa*	[343] 2019
Natamycin	Zein Chitosan	Natamycin (5 mg) and zein (60 mg) in 70 *v*/*v*% ethanol (20 mL) Carboxymethyl chitosan (CMCS, DA 95%, carboxylation degree of 70%) in water	Nanoprecipitation by anti-solvent method	Completely inhibited spore germination rate, inhibited mycelial growth by 64.4%	[344] 2020
	Lecithin Chitosan	Lecithin (2.5% *w*/*v*) and natamycin (0.2% *w*/*v*) in methanol Chitosan (DA 75–85%, MW 50–190 kDa, 1% *w*/*v*) Lecithin/chitosan ratio (20:1, 10:1, and 5:1, *w*/*w*)	Ionic gelation	Against *C. albicans* and *A. fumigates*	[345] 2012
	Gliadin Cellulose	Gliadin and natamycin in 70% ethanol Sodium CMC in water	Nanoprecipitation by anti-solvent method	Against *P. expansum*	[346] 2023
	Chitosan	Chitosan (DA 96.1%, MW 12 kDa, 1% *w*/*v*) TPP Volume ratio of chitosan to TPP (5:1) Natamycin (1 mg/mL) Volume ratios of natayicin to chitosan and TPP (1:20, 1:12, 2:15, 1:6, 1:4, and 1:3)	Ionic gelation	Strong antifungal effect on *C. albicans*	[347] 2021
	Alginate	Natamycin solution (20 mg/mL ethanol) Sodium alginate (0.3 mg/mL) Pluronic F-127 (10% *w*/*v*) Calcium chloride (0.67 mg/mL)	Emulsification followed by ionic gelation	*A. flavus* count was reduced by 2 log	[348] 2021

Notes: DA—deacetylation degree; MW—molecular weight; TPP—sodium tripolyphosphate; BNC—bacterial nanocellulose; CNFs—cellulose nanofibers; DS—degree of substitution; TONFC—2,2,6,6-tetramethyl-1-piperidinyloxyl-oxidized nanofibrillated cellulose; HCNF—holocellulose nanofibrils; DAC—dialdehyde HCNF; HMP—high-methoxyl pectin; LMP—low-methoxyl pectin; DE—degree of esterification; HMPOS—high methoxy pectin oligosaccharide; NHS—N-hydroxysuccinimide; EDC—1-(3-dimethylaminopropyl)-N’-ethyl-carbodiimide hydrochloride; CMCS—carboxymethyl chitosan; CMC—carboxymethylcellulose.

**Table 5:** Polysaccharide-based nanostructures incorporating animal-derived antimicrobial agents.

Active Component	Nanocarrier	Composition	Formulation Method	Antimicrobial Activity	Ref. and Pub. Year
Lysozyme (Ly)	Chitosan	Chitosan (MW 50–100 kDa, 0.5% *w*/*v*) TPP (0.25%, *w*/*v*) Chitosan-to-TPP ratio (3:1 *v*/*v*) Ly (0.5% *w*/*v*, 0.25, 0.50, 0.75, 1.00, and 1.25 mg/mL)	Ionic gelation	Against *E. coli* and *B. subtilis*	[373] 2017
		Chitosan (DA 78%, MW 153 kDa, 0.5 mg/mL *w*/*v*) Ly (≥40, 000 U/mg protein, 0.5 mg/mL *w*/*v*) Acetone Tween 80 (0.05% *v*/*v*)	Nanoprecipitation	Reduced viability of *A. parasiticus* and strongly inhibited spore germination	[374] 2017
		Chitosan (DA 93%, 1 mg/mL *w*/*v*) TPP (1 mg/mL) Ly (10 mg/mL) EDC and NHS (0.1 M)	Ionic gelation followed by conjugation	Inhibited growth of *S. aureus*, *E. coli*, *P. aeruginosa*, and *K. pneumoniae*	[375] 2020
		Carboxymethyl chitosan (CMC) Ly (from chicken egg whites) Amorphous calcium phosphate (ACP)	Polyelectrolyte complexation	Against *S. mutans*	[376] 2025
	Chitosan Alginate	Sodium alginate (MW 140 kDa, mannuronate/guluronate ratio 1:1, 0.5 mg/mL) Ly (from eggs, 40,000 U/mg) Chitosan (DA ≥ 80%) Calcium chloride (1.0, 3.0 or 5.0 mM) Chitosan/sodium alginate mass ratio (1:2) Polymer-to-ly mass ratio (10:1)	Alginate pre-ionic gelation followed by chitosan coacervation	No data	[377] 2018
	CNCs	High MW chitosan (DA 88.24%, MW 1400 kDa, 0.25 mg/mL) Low MW chitosan (DA 86.39%, MW 45.25 kDa, 0.25 mg/mL) CNCs (0.5% *w*/*v*) Ly (1.00, 1.25, 1.50, 1.75 and 2.00 mg/mL)	Complexation	Against *E. coli* and *L. innocua*	[378] 2020
	Chitosan Dextran	Depolymerized chitosan (DA 89%, 0.1% *w*/*v*) Dextran sulfate (MW 500 kDa) Ly Zinc sulfate solution (1 M)	Polyelectrolyte complexation	No data	[379] 2019
	Alginate	Sodium alginate (low viscosity, 4% *w*/*v*) Ly nanofibers (LNFs) from hen egg white ly (~70,000 U/mg) LNFs (1, 5 and 10 wt.% with respect to the alginate mass) Calcium chloride (0.5% *w*/*v* and 2% *w*/*v* for finilazing) Alginate-LNFs:calcium chloride volume ratio (4:1)	Ionic gelation		[380] 2022
Lysozyme (Ly)	CNCs	Enzymatic neutral CNCs (N_CNCs) Sulfated CNCs (S_CNCs) Ly from chicken egg white (≥40,000 U/mg protein EDC and NHS (1:1 weight ratio) Incubation Ly and nanocrystals (1 : 10 weight ratio)	Covalent and nonspecific immobilization	Against *M. deykticus*	[381] 2024
		CNCs (50 mg) Ly (2 or 4 mg/mL) CNC (1% *w*/*v*) EDC and NHS Amino-Functionalized CNC (2 g/mL) Glutaraldehyde (750 μL)	Physical adsorption and covalent immobilization	Against *M. lysodeikticus*, *Corynebacterium* sp., *E. coli*, and *Ps. mendocina*	[382] 2017
	CA Nanofibers Pectin	Cellulose acetate (CA, MW 30 kDa) Pectin (from citrus fruits) Ly (25,000 U/mg, 1 mg/mL)	Electrostatic adsorption in layer-by-layer assembly	Against *E. coli* and *S. aureus*	[383] 2015
	Starch	Potato starch 2,2,6,6-tetramethyl1-piperidinyloxy (TEMPO) Sodium trimetaphosphate (STMP) Ly (from chicken egg white, 50 mg/mL) Cross-linker to polymer weight ratio (0.10—0.40)	Cross-linking and Ly absorption	Against *B. licheniformis* 7558, *B. licheniformis* 6993, *B. subtilis* 168, *L. monocytogenes* LR991, and *L. monocytogenes* 001	[384] 2012
		Potato starch Ly (from chicken egg white, ≥98%) STMP (≥99%, 0.1 g/mL) Cross-linker to polymer weight ratio (0.1, 0.25 and 0.4) N-hexane Ly (25 mg, 0.625 mg/mL) Bacillus licheniformis protease (13.7 U/mg) Ly and hydrolyzed amphiphilic peptides formed Ly NP (0.15, 0.30, 0.60, 1.0, 1.25, 2.5, 5 and 10 mg/mL)	Water-in-oil emulsification with cross-linking, followed by electrostatic adsorption of self-assembled LNP	No data	[385] 2020
	Pectin	Low-methoxyl amidated pectin (DE 22–28%, DAc 20–23%, 0.05 g/L) Lysozyme (from hen egg white, 70,000 units/mg, 0.714 g/L) Calcium chloride (0–30 g/L)	Ionic gelation	Against *M. lysodeikticus*	[386] 2014
		Pectin (galacturonic acid (dry basis) ≥ 74.0%, 0.5 mg/mL or 1 mg/mL) Ly (>20 ku/mg, 0.5 mg/mL) Ly-to-polymer ratio (1:1 or 1:2)	Complex coacervation via electrostatic interactions	No data	[387] 2021
	Gum Arabic (GA)	Gum arabic (GA) Ly (from chicken egg white) Ly and GA in weight ratio (1:1, 1:2, and 1:4, 200:800 mg)	Complex coacervation via electrostatic interactions, followed by Maillard reaction-mediated conjugation	Against *E. coli* and *S. aureus*	[388] 2018
	Carrageenan	κ-carrageenan (CRG) Ly Mass ratio of Ly to CRG (3:1, 2:1, or 1:1) Curcumin (2.5 mg/L, 7.5 mg/L, or 12.5 mg/L)	Complex coacervation	No data	[389] 2020
Lysozyme (Ly)	Xanthan Gum (XG)	Xanthan gum (XG, 1.0 mg/mL) Ly (from chicken egg white, 1.0 mg/mL) Weight ratios (3:1, 2:1, 1:1, 1:2, and 1:3)	Complex coacervation via electrostatic complexation		[390] 2015
		Xanthan gum (XG, MW 3000–20,000 kDa, 1.0 mg/mL) Ly (from chicken egg white, 1.0 mg/mL) Ly/XG weight ratios (2:1, 1:1, and 1:2) In alkaline conditions	Complex coacervation via electrostatic complexation, followed by gelatinization under alkaline conditions		[391] 2018
		Xanthan gum (XG) Ly (from chicken egg white) Ly/XG weight ratios (2:1, 1:1, and 1:2) In alkaline conditions			[392] 2018
		Xanthan gum (XG) Ly (from chicken egg white) XG/Ly ratios (4:1, 1:1, and 1:4)	High-pressure homogenization (HPH)-assisted electrostatic complexation		[393] 2021
	Dextran	Dextrans (MW 10, 35, and 62 kDa) Ly (from hen egg white) Molar ratios of dextran to Ly (1:8, 1:4, 1:2, 1:1, 2:1, 4:1, and 8:1)	Conjugation via Maillard dry heating and thermal gelation	No data	[394] 2008
		Dextrans (MW 60–90 kDa) Ly (from fresh egg white) Mass ratio of Ly to dextran (1:5)	Conjugation via Maillard dry heating	Against *M. Lysodeikticus*, *V. parahaemolyticus* IFO 13286, *E. coli* IFO 12713, *A. hydrophila* IFO 13286, *P. mirabilis* IFO 12668, *K. pneumoniae* IFO 14438, *B. cereus* IFO 13690, and *S. aureus* IFO 14462.	[395] 1991
	Pullulan	Pullulan (MW 20 kDa) Ly (from chicken egg white) Ly-to-pullulan molar ratios (1:2, 1:4, 1:6, 1:8, 1:10, and 1:12)		Against *E. coli*, *S. enterica*, and *S. aureus*	[396] 2017
		Dialdehyde pullulan polysaccharide (MW 200 kDa) Ly	Electrospinning followed by cross-linking with Ly	Against *E. coli* and *S. aureus*	[397] 2025
	Hyaluronic Acid (HA)	HA (MW 870 kDa, 0.768 mg/mL to 0.144 mg/mL) Ly (from hen egg white, 2.6 mg/mL) HA:Ly ratio of 1:5 (*v*/*v*)	Complex coacervation	No data	[398] 2014
		HA (MW 1000–1800 kDa, 2%) Ly (from egg white, 6%) Volume ratio (1:1)		Promoting wound healing	[399] 2020
Lactoperoxidase (LPO) and lactoferrin (LF)	Chitosan Dextran	Chitosan Dextran sodium sulfate LPO and LF	Complex coacervation	No data	[400,401] 2023 2022
		Chitosan (2 mg/mL) Dextran sodium sulfate LPO and/or LF (0.5 mg/mL)			[402] 2017
LPO	Chitosan Gum Tragacanth	Chitosan (0.005%) Gum tragacanth (0.005%) Tragacanth/chitosan ratio (1:8) Lactoperoxidase (LPO, 80 U/mL)			[403] 2015
LF	Chitosan	Chitosan (DA 75–85%, MW 150 kDa, 0.045 and 0.055% *w*/*v*) LF (96% *w*/*w*, 0.035 and 0.045% *w*/*v*) TPP (0.01% *w*/*v*) LF/chitosan/TPP ratios (3.5:5.5:1 and 4.5:4.5:1)	Ionic gelation	Against *S. aureus*	[404] 2023
		Chitosan (DA 85%, MW 150 kDa, 0.05–0.2% *w*/*v* for TPP and 0.025 to 0.1% *w*/*v* for SBE-β-CD) TPP (0.05–0.2% *w*/*v*) Chitosan/TPP ratio (5:1 *v*/*v*) Sulfobutylether-β-cyclodextrin (SBE-β-CD, MW 2160 Da, substitution degree = 3.00–6.50, 0.1 to 0.5% *w*/*v*) LF (from 0.1 to 1.0 mg/mL)		No data	[405] 2021
	Chitosan Gellan Gum	Chitosan (DA 75–85%, MW 150 kDa, 0.045% *w*/*v*) Gellan gum (0.01% *w*/*v*) LF (0.045% *w*/*v*) LF/chitosan/gellan gum ratio (4.5:4.5:1)	Electrostatic complexation	Against *S. aureus*	[406] 2022
	Alginate	Sodium alginate solution (0.2 and 0.5% *w*/*w*) LF (0.1%) Glycerol (27% *v*/*v*) and tween 156 80 (4% *w*/*v*) Calcium chloride (0.5% *w*/*v*)	Oil-in-water emulsification followed by ionic gelation	No data	[407] 2015
		Sodium alginate solution (0.2 and 0.5% *w*/*w*) LF Calcium chloride	Ionic gelation		[408] 2022
	BNC	BNC, oxidized BNC LF (0.25, 0.5, 1 and 2 mg/mL)	Absorption or covalent binding	Against *S. aureus* and *E. coli*	[409] 2020
	Gellan Gum	Gellan gum (0.01–0.08% *w*/*v*) LF stock solution (0.01–0.09% *w*/*v*) LF/gellan gum ratios 2:8, 5:5, 6:4, 7:3, 8:2, 9:1	Electrostatic complexation	Against *S. aureus* and *E. coli*	[410] 2022
	Pectin	Pectin LF Ciprofloxacin and naringin	Complex coacervation	Against *P. aeruginosa*	[411] 2024
		Pectin (galacturonic acid ≥ 74%, methoxyl groups ≥ 6.7%, MW 1900 kDa) LF (1.0 mg/mL) Ratio 1:1 (*w*/*w*)		No data	[412] 2017
	Hyaluronic Acid (HA)	Hyaluronic acid (HA, MW 9.8 kDa, 0.52 wt% and 0.4 wt% for covalent conjugation) LF (0.5 wt% and 1.0 wt% for covalent conjugation) EDC and NHS EDC:NHS:HA (1:1:1 mole ratio)	Non-covalent and covalent conjugation	No data	[413] 2021
Ovotransferrin (OVT)	Gum Arabic	Gum arabic (GA, 1 wt%) OVT (purity > 88%, 3 wt%) Sodium citrate buffer	Complexation	No data	[414] 2019
		Gum arabic (GA, 1 wt%) OVTFs (purity > 88%, wt%) Sodium chloride Total biopolymer concentration of 50 mg/mL (equal proportions of polymers)			[415] 2024
	Chitosan	Carboxymethyl chitosan (CMCS, degree of carboxylation ≥ 80%, 20 mg/mL) Ovotransferrin (OVT, purity > 88%, 60 mg/mL) Equal volumes of OVT and CMCS	Complexation	No data	[416] 2022
	Pectin	Citrus pectin (CP) Ovotransferrin fibrils (OVTFs) OVTF-to-CP mass ratio of 3:1	Electrostatic attractions		[417] 2023
		Sugar beet pectin (SBP, DE 55%, MW 65 kDa, 0.1 mg/mL) OVT (purity > 88%, 0.05–0.5 mg/mL) OVT/SBP mass ratios (from 1:2 to 5:1)	Complexation		[418] 2019
Cryptdin-2	Chitosan	Chitosan (DA 75–85%, medium MW, 0.1%, 0.5%, 1%) TPP (0.1%, 0.5%, 1% *w*/*v*) Ratio of chitosan to TPP (5:2, 5:1, 1:1) Cryptdin-2 (1 mg/mL)	Ionic gelation	2 log unit reductions in *Salmonella Typhimurium* load in mice tissues	[419] 2015
Temporin B		Chitosan (DA 92%, MW 108 kDa, 1 mg/mL) TPP (1 mg/mL) Ratio of chitosan to TPP (5:2) Temporin B (200 μg)		Up to 4-log reduction in number of viable *S. epidermidis*	[420] 2015
Cecropin-B		Chitosan (low MW, 2 mg/mL) TPP (1 mg/mL) Weight ratio TPP to chitosan (1:2) Cecropin-B (50 µg/mL)		Against multidrug-resistant *K. pneumoniae*	[421] 2023
Defensin HNP-1		Chitosan (low MW, 4 mg/mL) TPP (4 mg/mL) Defensin HNP-1 (0.2/ mL)		Against *S. aureus* ATCC 25923, *E. coli* NCTC 9001, *P. aeruginosa* ATCC 10145, *K. aerogenes* NCTC 10006, and MRSA	[422] 2021
Human cathelicidin peptide (LL-37)		Chitosan (DA 95%, MW 100–300 kDa) TPP LL-37		Against MRSA	[423] 2022
Pleurocidin-like peptide NRC-07		Chitosan (low MW, 0.5% *w*/*v*) TPP (0.125% *w*/*v*) NRC-07 (1 mg) Volume ratio of TPP to chitosan (1:1)		Against *P. aeruginosa*	[424] 2024
Cecropin CA(1–7)M(2–9)	CNFs	CNFs (0.1%) CA(1–7)M(2–9) (6.5 and 13 mg/mL)	Immobilization	Against *B. subtilis*	[425] 2017
Antimicrobial motif of LL-37 (KR-12)		CNFs KR-12 Cross-linkers for carbodiimide chemistry, thiol-ene click chemistry, and Cu(I)-catalyzed azide-alkyne cycloaddition	Conjugation	Against *E. coli* and *S. aureus*	[426] 2023
Minimal motif of bovine lactoferricin (LfcinB) (20–25)_Pal_	Pullulan	Pullulan (200 kDa, 20% *w*/*w*) LfcinB (20–25)Pal peptide (13.2 mg/mL) Pullulan/peptide 74:1 *w*/*w*	Electrospinning	Against *E. coli*	[427] 2019
Antimicrobial motif of LL-37 (KR-12)	Starch	Potato starch (St) TEA (0.6 mL) Norbornene anhydride (1.5 g, 0.3 equivalent to −OH group of one unit, degree of substitution (DS) of St = 30%) DMAP (1.1 g) Dithiol-functionalized poly (ethylene glycol) (HS-PEG-SH)/modified St molar ratios (3:1 and 3:2) Photoinitiator Irgacure 2959 (0.5%) Cys-KR12 (100 μL, 1 mg/mL)	Immobilization	Against *S. aureus*, *S. epidermidis*, *E. coli*, and MRSA	[428] 2019
Snake cathelicidin Ab-Cath	Hyaluronic Acid	Octenyl succinic anhydride-modified hyaluronic acid (OSA-HA, 17–32% degree of substitution, MW 50 kDa, 500 μg/mL) Ab-Cath (1500 μg/mL, 10× final peptide concentration) Cryoprotectant solution	Microfluidic chip design	Against *S. aureus*, *A. baumannii*, and *E. coli* in biological fluids; reduced *S. aureus* and *A. baumannii* biofilms	[429] 2023
Thanatin		Hyaluronic acid (HA) PLGA Thanatin HA/PLGA/thanatin ratio of 1:1:0.8	Complexation	Against sepsis induced by metallo-β-lactamases-1 (NDM-1) producing *E. coli*	[430] 2025
Cecropin B		Hyaluronic acid (HA) Cecropin B	Electrostatic interaction in water	No data	[431] 2023

Notes: DA—deacetylation degree; MW—molecular weight; TPP—sodium tripolyphosphate CMC—carboxymethyl chitosan; ACP—amorphous calcium phosphate; NHS—N-hydroxysuccinimide; EDC—1-(3-dimethylaminopropyl)-N’-ethyl-carbodiimide hydrochloride; CNCs—cellulose nanocrystals; LNFs—lysozyme nanofibers; CA—cellulose acetate; TEMPO—2,2,6,6-tetramethyl1-piperidinyloxy; STMP—sodium trimetaphosphate; DE—degree of esterification; XG—xanthan gum; GA—gum arabic; CRG—κ-carrageenan; Ly—lysozyme; HA—hyaluronic acid; LPO—lactoperoxidase; LF—lactoferrin; SBE-β-CD—sulfobutylether-β-cyclodextrin; BNC—bacterial nanocellulose; CMCS—carboxymethyl chitosan; OVT—ovotransferrin; OVTFs—ovotransferrin fibrils; CP—citrus pectin; SBP—sugar beet pectin; DMAP—4-Dimethylaminopyridine; TEA—triethanolamine; HS-PEG-SH—dithiol-functionalized poly (ethylene glycol); OSA-HA—octenyl succinic anhydride-modified hyaluronic acid; PLGA—poly(lactic-co-glycolic acid).

## Data Availability

Data sharing is not applicable.

## Abbreviations

The following abbreviations are used in this manuscript:

PLA	Polylactic acid
PHA	Polyhydroxyalkanoates
PLGA	Poly(lactide-co-glycolide)
PCL	Poly(caprolactone)
TPS	Tea water extract
DA	Deacetylation degree
MW	Molecular weight
CEO	Clove essential oil
GLEO	Guava leaves essential oil
TPP	Sodium tripolyphosphate
MSSA	Methicillin-susceptible *S. aureus*
EDC	Ethylene carbo-di-imide hydrochloride
NHS	N-hydroxysuccinimide
MDR	Multidrug-resistant
EO	Essential oil
BNC	Bacterial nanocellulose
CA	Cellulose acetate
PM	Peppermint
CN	Cinnamon
LG	Lemongrass
CNFs	Cellulose nanofibrils
DCC	N, N’-dicyclohexylcarbodiimide
DMAP	4-Dimethylaminopyridine
TC	*Triphala Churna*
MRSA	Methicillin-resistant *S. aureus*
CPE	Citrus peel extracts
ANCs	Anthocyanins
MD	Methylation degree
PAGE	*Prunus armeniaca* gum exudates
DL	D-limonene
GCN	Grapefruit seed extract and cinnamon oil
SPu	Succinylated pullulan
HA	Hyaluronic acid
OLE	Olive leaf extract
SF	Silk fibroin
TEA	Triethanolamine
DMTMM	4-(4,6-dimethoxy-1,3,5-triazin-2-yl)-4-methylmorpholinium chloride
3-APBA∙HCl	3-aminophenylboronic acid hydrochloride
DS	Degree of substitution
TONFC	2,2,6,6-tetramethyl-1-piperidinyloxyl-oxidized nanofibrillated cellulose
HCNF	Holocellulose nanofibrils
DAC	Dialdehyde HCNF
HMP	High-methoxyl pectin
LMP	Low-methoxyl pectin
DE	Degree of esterification
HMPOS	High methoxy pectin oligo-saccharide
CMCS	Carboxymethyl chitosan
CMC	Carboxymethylcellulose
AMP	Antimicrobial peptide
CNCs	Cellulose nanocrystals
LNFs	Lysozyme nanofibers
TEMPO	2,2,6,6-tetramethyl1-piperidinyloxy
STMP	Sodium trimetaphosphate
XG	Xanthan gum
GA	Gum arabic
CRG	κ-carrageenan
Ly	Lysozyme
LPO	Lactoperoxidase
LF	Lactoferrin
SBE-β-CD	Sulfobutylether-β-cyclodextrin
OVT	Ovotransferrin
OVTFs	Ovotransferrin fibrils
CP	Citrus pectin
SBP	Sugar beet pectin
HS-PEG-SH	Dithiol-functionalized poly (ethylene glycol)
OSA-HA	Octenyl succinic anhdride-modified hyaluronic acid
